# Adipose-derived mesenchymal stromal cells promote corneal wound healing by accelerating the clearance of neutrophils in cornea

**DOI:** 10.1038/s41419-020-02914-y

**Published:** 2020-08-26

**Authors:** Qianwen Shang, Yunpeng Chu, Yanan Li, Yuyi Han, Daojiang Yu, Rui Liu, Zhiyuan Zheng, Lin Song, Jiankai Fang, Xiaolei Li, Lijuan Cao, Zheng Gong, Liying Zhang, Yongjing Chen, Ying Wang, Changshun Shao, Yufang Shi

**Affiliations:** 1 The First Affiliated Hospital of Soochow University, State Key Laboratory of Radiation Medicine and Protection, Institutes for Translational Medicine, Soochow University Medical College, Suzhou, Jiangsu 215123 China; 2 Department of Ophthalmology, The Affiliated Hospital of Jiangnan University, 200 Huihe Road, Wuxi, 214062 China; 3 The Second Affiliated Hospital of Soochow University, Suzhou, Jiangsu 215123 China; 4 Key Laboratory of Stem Cell Biology, Shanghai Jiao Tong University School of Medicine and Shanghai Institutes for Biological Science, Chinese Academy of Sciences, Shanghai, 200025 China

**Keywords:** Chronic inflammation, Stem-cell research

## Abstract

The dome-shaped cornea is a transparent, non-vascularized, and epithelialized highly organized tissue. Physical and chemical injuries may trigger corneal wound healing (CWH) response and result in neovascularization that impairs the visual function. CWH involves not only migration, proliferation, and differentiation of the cells in different layers of cornea, but also the mobilization of immune cells. We demonstrated here that human adipose-derived mesenchymal stromal cells (ADSCs) could effectively inhibit neovascularization during ethanol-induced injury in mouse cornea. Importantly, we found that while neutrophils are essential for CWH, excessive and prolonged neutrophil retention during the granulation stage contributes to neovascularization. ADSCs were found to promote the clearance of neutrophils in the cornea during the granulation stage, likely via increasing the reverse transendothelial cell migration of CXCR4^high^ neutrophils from cornea to the lung. Our results demonstrate that ADSCs are effective in treating CWH-induced neovascularization and modulation of neutrophil clearance could be novel strategies for better vision recovery after injury.

## Introduction

In addition to protecting the interior structure of the eye from environmental insults, the dome-shaped cornea also acts as a lens to focus light entering the eye. The latter function of the cornea depends on its precise radians and flawless transparency. However, trauma, burns, and infections may cause injury to the cornea and subsequently trigger corneal wound healing (CWH) response. Because CWH is often accompanied by disorganized structure of vague collagenous fibers and corneal neovascularization, corneal transparency may be compromised after healing. Thus the avascular status of the cornea is essential for its visional function and needs to be conscientiously maintained^1^. As in skin wound healing, inflammation, granulation, and remodeling are the three major steps in the process of corneal repair^2–4^. Previous studies suggest that each of these steps is closely associated with neovascularization^5–9^, however, the kinetics of this process is not fully described. Therefore, a better understanding of the pathophysiological process involved in CWH can help to identify the key steps involved in corneal neovascularization.

Mesenchymal stromal/stem cells (MSCs) are multipotent and can be readily isolated and expanded from a number of tissues, including bone marrow, fat tissue, Wharton’s jelly of the umbilical cord, and amniotic fluid^10^. MSCs contribute to tissue homeostasis and regeneration by providing progenitor cells, growth factors, and most importantly the modulation of the inflammatory responses. This immunomodulatory ability of MSCs makes them a promising candidate for cell-based therapy in diverse types of tissue damages^11,12^. It has been reported that rat bone marrow-derived MSCs can inhibit corneal neovascularization induced by chemical injury^13^. Subsequent study has shown that human bone marrow MSCs (hBMMSCs) could induce tolerance against allo- and autoimmunity in the eye^14^. Most recently, it was reported that hBMMSCs inhibit lymphangiogenesis in the cornea by suppressing the recruitment of inflammatory macrophages^15^. These studies suggest a potential role of MSCs-based therapy in alleviating corneal neovascularization and implicated the importance of immune cells in neovascularization during CWH. As neutrophils are among the first responders to wound sites, neutrophil infiltration during CWH were thought to play a critical role in the healing process^16^. Neutrophils are thought to be important in angiogenesis, through releasing pro-angiogenic factors^17^. Moreover, MSCs have been reported to regulate neutrophil migration^18^. However, whether neutrophils play a role in corneal repair and neovascularization and if so, whether their functions in CWH can be modified by MSCs remain largely unknown.

Compared with BMMSCs, adipose-derived mesenchymal stem/stromal cells (ADSCs) are relatively easier to obtain and also possess immunosuppressive and pro-regenerative properties^19–22^. They are 100 times more abundant in adipose tissue than MSCs in the bone marrow^23^. These properties make ADSCs favorable candidates in clinical applications. Indeed, injection of ADSCs resulted in better and faster re-epithelization and less inflammatory response in corneal lesions^24^. In this study, we sought to determine whether ADSCs expanded in vitro can be employed to promote the healing of corneal injury and, if so, whether such effect is related to their regulation of infiltration and clearance of neutrophils in cornea.

We established the ethanol-induced corneal injury mouse model and tested the effect retro-orbital injection of human ADSCs. Our results revealed that ADSCs dramatically reduced corneal neovascularization induced by ethanol exposure. This effect is achieved through inhibiting the formation of ECM during granulation. ADSCs treatment also promotes the reverse migration of CXCR4^high^ neutrophils from ocular tissue and thereby alleviates neovascularization, supporting a role of excessive neutrophil retention in neovascularization during the granulation stage. Together, our results demonstrated an antiangiogenic function of ADSCs during CWH and that promoting the reverse migration of neutrophils could be a new approach for the treatment of corneal injury.

## Materials and methods

### Animals

BALB/c male mice were purchased from Beijing Vital River Laboratory Animal Technology Co. Ltd. (Beijing, China), and maintained under specific pathogen-free conditions. All procedures were approved and conducted under the Guideline for the Institutional Animal Care and Use Committee of Soochow University.

### Cell isolation and culture

ADSCs were obtained from the adipose tissue of lipoaspirate samples following the protocols approved by the Ethics Committee of Soochow University. Informed Consent was obtained from each patient. Adipose tissues were washed extensively with phosphate-buffered saline (PBS) to remove debris and then treated with 0.1% collagenase I in DMEM/F12 medium with 10% fetal bovine serum (FBS) for 1 h at 37 °C with gentle agitation. The digested samples were centrifuged at 400*g* for 5 min to separate adipocytes from stromal vascular fraction. The cell pellet containing the stromal fraction was resuspended in DMEM/F12 supplemented with 10% FBS, 100 U/mL penicillin/streptomycin solution and 10 ng/mL bFGF and cultured in 10 cm dishes at 37 °C in a humidified atmosphere with 5% CO_2_. The medium was changed every 3 days thereafter. When the cells reached 90% confluence, the cultures were trypsinized and re-passaged. Passage 3–9 cells were used for the described experiments. ADSCs were characterized by flow cytometry (Beckman Cytoflex) with a series of primary antibodies described in Supplementary Fig. 1A. Adipogenic and osteogenic differentiation of ADSCs were assessed as previously described^25^.

### Corneal injury healing model

Mice were anesthetized and the right eyes were immersed in 75% ethanol for 60 s, and were then immediately flushed with 10 ml PBS. For ADSC treatment, 5 × 10^5^ cells or equal volume (75 μL) PBS were injected into the retro-orbital area 1 h after ethanol injury. Antibodies and inhibitors were in 100 μL PBS and intraperitoneally injected. Photos were taken under an anatomic microscope (Nikon SM27457) at different time points after treatment. Image pro plus 6.0 was used for the statistic analysis of the vascular area with the double blind method.

### Fluorescein cornea angiography

Fluorescein sodium (2.5 μg/mice) was intravenously injected. After 1 min, the mice were euthanized and their corneas were removed immediately. The fluorescence was stimulated by blue illuminant and photographically recorded under an anatomic microscope (Nikon SM27457).

### Sirius red stain and collagen quantification

Picro-sirius red staining kit (PSR-1, ScyTek) was used to visualize collagen fibrils. The total collagen content in the tissue sections were measured by Sirius Red/Fast Green Collagen Staining Kit (9046, Chondrex) according to the manufacturer’s instructions.

### Hematoxylin-eosin staining and immunofluorescence

The eyeballs were fixed in 4% paraformaldehyde for 24 h at room temperature, dehydrated with 30% sucrose before transferred into OCT medium (4583, SAKURA) and then frozen at −20 °C. The tissues were sectioned into 8 μm in thickness and placed on glass slides. The tissues mounted on slides were then washed with ddH_2_O for 10 min to remove OCT. For hematoxylin-eosin staining, slides were stained with hematoxylin (MINDEL GTS-1096) for 5 min, then stained with eosin (G1100, Solarbio) for 90 s. For immunofluorescence staining, slides were treated with 0.5% Triton (V900502-100ML, Sigma) for 15 min followed by 10% donkey serum for 1 h before incubating with primary antibodies overnight at 4 °C. On the next day, sections were washed with 0.1% Tween 20 in PBS (PBST) and incubated with the secondary antibodies for 1 h at room temperature. The nuclei were stained with Hoechst 33324 (H3570, Thermo Fisher Scientific). The antibodies (Abcam) against CD31, α-smooth muscle actin, Ly6G and MPO were used as primary antibodies. Secondary antibodies were Alexa 488-conjugated-goat anti-rabbit IgG (Abcam), Alexa 594-conjugated-goat anti-rat IgG (Abcam), and Alexa 555-conjugated-goat anti-mouse IgG (Thermo Fisher Scientific). Images were captured under a laser-scanning confocal microscope (Leica TCS SP8, Leica).

### Flow cytometry analysis

Whole eyeball cell suspensions were prepared following digestion with dispase and collagenase I as described for CFs isolation and red blood cells (RBC) were removed using a lysis solution (Invitrogen/Life Technologies), and washed twice in PBS (GIBCO) with 2% FBS. Lung tissues were cut and digested with collagenase II (0.5 mg/mL, 17101015, Thermo Fisher Scientific) for 1 h at 37 °C. Single-cell suspension of the lungs was prepared by pressing digested tissue through a cell strainer (70 µm) followed by RBC lysis. Cells were labeled with the monoclonal antibodies as described in the Reagent List (Supplementary Table 1).

For flow cytometric analysis, cells were stained with antibodies at 1:500 in 5% FBS/PBS for 30 min on ice. All flow cytometric analyses were performed using the Cytoflex Flow Cytometer (Beckman Coulter). Data were analyzed using the Flow Jo software (Version 9.6.2, Tree Star Inc.).

### Culture of HuVEC in ADSCs-conditioned medium

ADSCs were cultured in DMEM/F12 supplemented with 10% FBS, 100 U/mL penicillin/streptomycin at 37 °C in a humidified atmosphere with 5% CO_2_. The condition medium was collected after 3 days of incubation. HuVECs (human umbilical derived endothelial cells, purchased from PromoCell (C-12203)) were plated in 6-well plates at a density of 3 × 10^5^ cells/well and cultured in complete Endothelial Cell Growth Medium (C-22010, PromoCell) or ADSCs conditional medium (1:1 with fresh medium) at 37 °C in a humidified atmosphere with 5% CO_2_ for 24 h. The cells were then collected for RNA extraction.

### Induction of NETs

Total neutrophils from mouse bone marrow were obtained by negative selection using the EasySep Mouse Neutrophil Enrichment Kit (19762, STEM CELL) according to the manufacturer’s specifications. Neutrophil extracellular traps (NETs) were isolated as previous described^26^. NETs formation was induced by overnight stimulation of neutrophils (10^7^ cells) with 20 nmol/L of phorbol 12-myristate 13-acetate (PMA). The NETs layer deposited at the bottom of the culture dish was washed twice in PBS and collected by vigorous pipetting with 6 mL DMEM high glucose medium (with 10% FBS). The cell debris was removed by centrifugation at 300*g* for 10 min and the supernatant containing NETs were used for experiments.

### Tube formation assay

Matrigel was 2× diluted (120 μL/well, 356234, BD Pharmingen) and added to wells of 48-well plates and placed at 37 °C for 3 h. Equal numbers of EAhy926 cells (as a gift from Prof. Quansheng Zhou) (3 × 10^4^ cell/well) suspended in DMEM high glucose medium (with 10% FBS) were seeded with or without NETs (200 μL) onto matrigel and incubated for 6 h at 37 °C. DNase1 (A610099-0250, BBI) were added at the concentration of 100 μg/μL. Images were captured using the cell imaging microporous plate detection system (Cytation5, BioTek).

### Depletion and mobilization of neutrophils

Systemic neutrophil depletion was achieved by i.p. injection of 200 µg of purified monoclonal anti-Ly6G (127649, Biolegend) in 200 μL PBS to each mouse every other day. For neutrophils mobilization, mice were injected i.p. with SB225002 (antagonist of CXCR2, 25 μg/200 μL, S7651, Selleck) or AMD3100 (antagonist of CXCR4,125 μg/200 μL, S8030, Selleck) every day from the 3^rd^ day to the 14^th^ day after injury. Control mice were injected with sterile PBS.

### Lung tissue explants culture

Lung tissues from each group of mice were chopped into small pieces. The explants were dispersed in DMEM supplemented with 10% FBS at half lung tissue per 500 μL, and incubated at 37 °C, 5% CO_2_ for 48 h. The supernatants from cultured lung explants were collected and centrifuged for ELISA.

### Enzyme-linked immunosorbent assay (ELISA)

Culture supernatants from lung tissue explant or mice serum were harvested for analyzing the production of CXCL12 using commercial ELISA kits (444207, Biolegend) following the manufacturer’s instructions.

### Western blotting analysis

Eyeball cells were lysed in RIPA buffer (P0013B, Beyotime) in the presence of protease inhibitors. Equal amounts of protein lysate samples were resolved on sodium dodecyl sulfate polyacrylamide gel electrophoresis and then transferred onto polyvinylidene fluoride membranes. After blocking with 5% bovine serum albumin in tris-buffered saline containing 0.1% Tween-20 for 2 h, the membranes were probed overnight at 4 °C with respective primary antibodies. Membranes were washed three times followed by incubation with horseradish peroxidase-conjugated secondary antibody for 1 h at room temperature. Signals were visualized by chemiluminescent substrate (Pierce Biotechnology, Rockford, IL) with a Super sensitive automatic imaging analysis system (Protein simple FluorChem HD2).

### Quantitative real-time PCR

Total RNA was extracted using RNAfast 2000 (Fastagen) and reversely transcribed into cDNA using a ReverTra Ace quantitative polymerase chain reaction (qPCR) RT Kit (TOYOBO Life Science) according to the manufacturer’s protocol. qPCR was carried out using SYBR Green Master Mix (4472920, Thermo Fisher Scientific). Primers used are listed in Supplementary Table 2. The relative mRNA levels of genes were calculated by the 2^−^^ΔΔCt^ method, using *β-actin* as internal control.

### In vivo imaging of ADSCs

ADSCs were suspended and incubated with 10 μM DiR buffer for 30 min at 37 °C according to the protocol from the manufacturer (125964, PerkinElmer). The DiR-labeled ADSCs were washed twice with PBS buffer and injected as above described. Mice were imaged dorsally with IVIS Spectrum on Day 1, 3, 7, an Day 14. The lungs were harvested for ex vivo imaging at different time points.

### Statistical analysis

One-way ANOVA was performed to determine statistical significance using GraphPad Prism 6. Values are presented as mean ± SEM. Differences were considered statistically significant at *P* values less than 0.05.

## Results

### ADSC treatment alleviates neovascularization in injured cornea

**Fig. 1:**
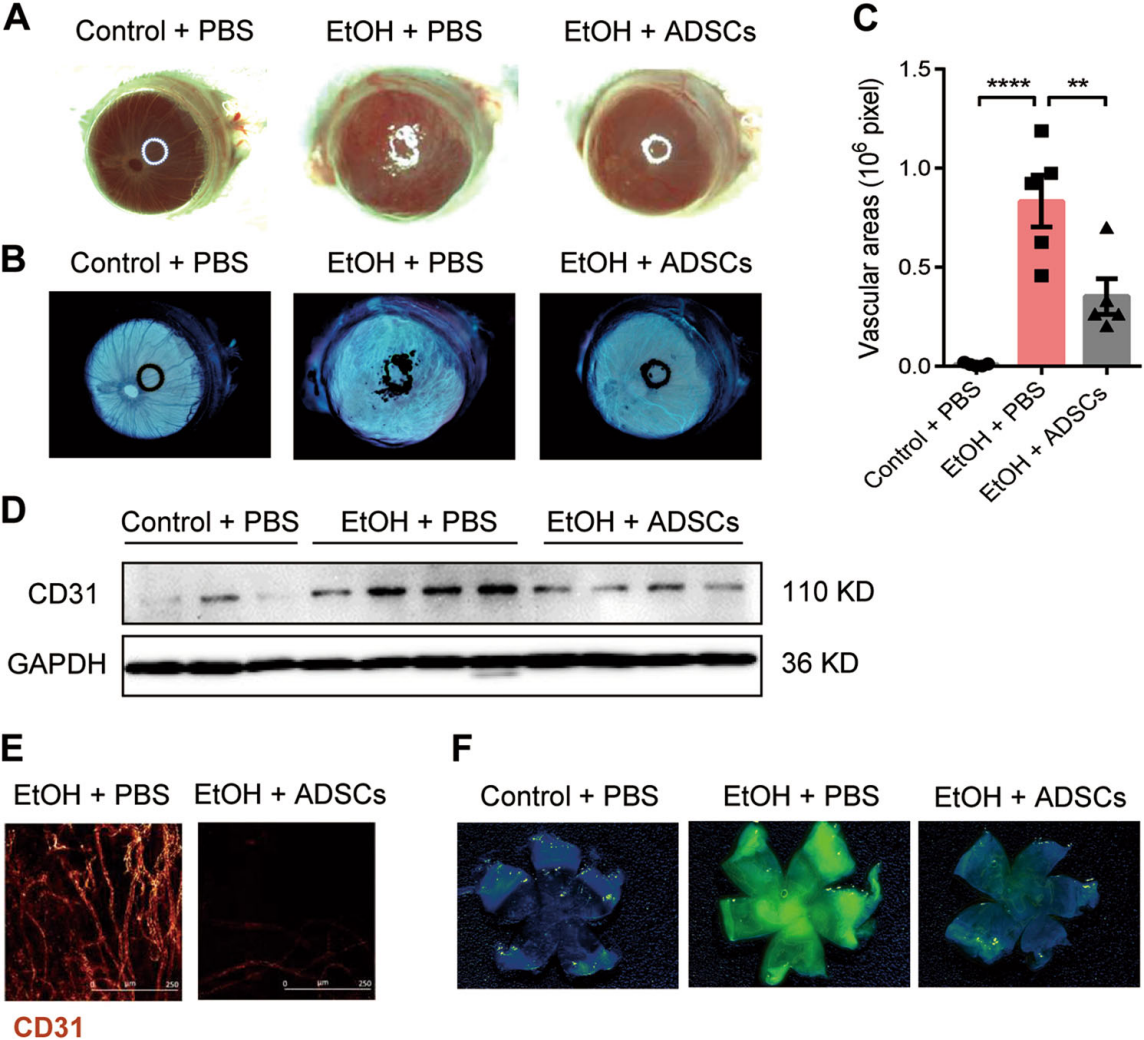
ADSC treatment alleviates neovascularization associated with corneal wound healing. **a**–**f** The eyes of male BALB/c mice were treated with or without (control group) 75% ethanol (EtOH & ADSCs group). The ADSCs group mice were injected with human ADSCs (5 × 10^5^ cells per mouse) 1 h after injury; control and EtOH groups treated with PBS were used as negative and positive controls. The bright-field photo of eyeballs (**a**) and their inverted phases (**b**) were taken in each group on Day 21. Vascular areas of corneas in each group (**c**) were presented as pixels (*n* = 4–5 mice/group). **d** The CD31 expression of the whole eyeball in each group was detect by Western blotting analysis, each band represents a different eyeball sample. The level of GAPDH in each sample was used as a loading control. **e** Immunofluorescence images of the whole cornea and the antibodies against CD31 was used to detect blood vessels. Scale bars represent 50 μm. Fluorescence photographs (**f**) of corneas in different groups were taken on Day 21, and sodium fluorescein (2.5 μg/mice) was injected into the tail vein before the methanation of the mice. Results are means ± SEM, *P < 0.05; **P < 0.01; ***P < 0.001 determined by one-way ANOVA with Tukey comparisons.

Like in most tissues, wound healing in cornea undergoes three typical stages: inflammation, granulation, and remodeling. Nevertheless, the repair process is usually accompanied by irreversible neovascularization, which often penetrate the stromal layer of the cornea and result in loss of corneal transparency. We, therefore, first explored the effect of ADSCs on CWH-induced neovascularization. Human ADSCs were isolated and characterized according to the standard protocol (Supplementary Fig. 1). To establish a chemical burn-induced corneal wound healing model, we covered the right eye of each mouse with 75% ethanol (EtOH) for 60 s and then flushed the eye with PBS immediately after. The EtOH-treated eyes were then treated within an hour with 5 × 10^5^ ADSCs or equal volume PBS via retro-orbital injection. The cornea in the ADSC treatment mice showed less cloudiness compared with the EtOH mice 28 days after injury (Fig. 1a). The area occupied by blood vessels in cornea was also significantly reduced after ADSCs treatment (Fig. 1b, c). In addition, the expression of CD31, representing endothelial cells, was markedly increased in the EtOH group but not in the EtOH + ADSCs group (Fig. 1d). To eliminate the interference of tunicae vasculosa, the whole cornea was removed for CD31 immunofluorescence staining and revealed tubular structures on the cornea of the EtOH group, whereas such structure is less apparent and exhibited weaker staining in the EtOH + ADSCs group (Fig. 1e). In addition, the fluorescein sodium staining of vascular endothelium showed that the EtOH group could emit intense green fluorescence due to neovascularization, while the fluorescence intensity in the EtOH + ADSCs group was much lower, indicating that the newly formed capillaries were reduced after ADSCs treatment (Fig. 1f). All these results suggest that ADSCs can significantly alleviate pathologic neovascularization in cornea.

### ADSC treatment reduces corneal thickening during granulation

**Fig. 2:**
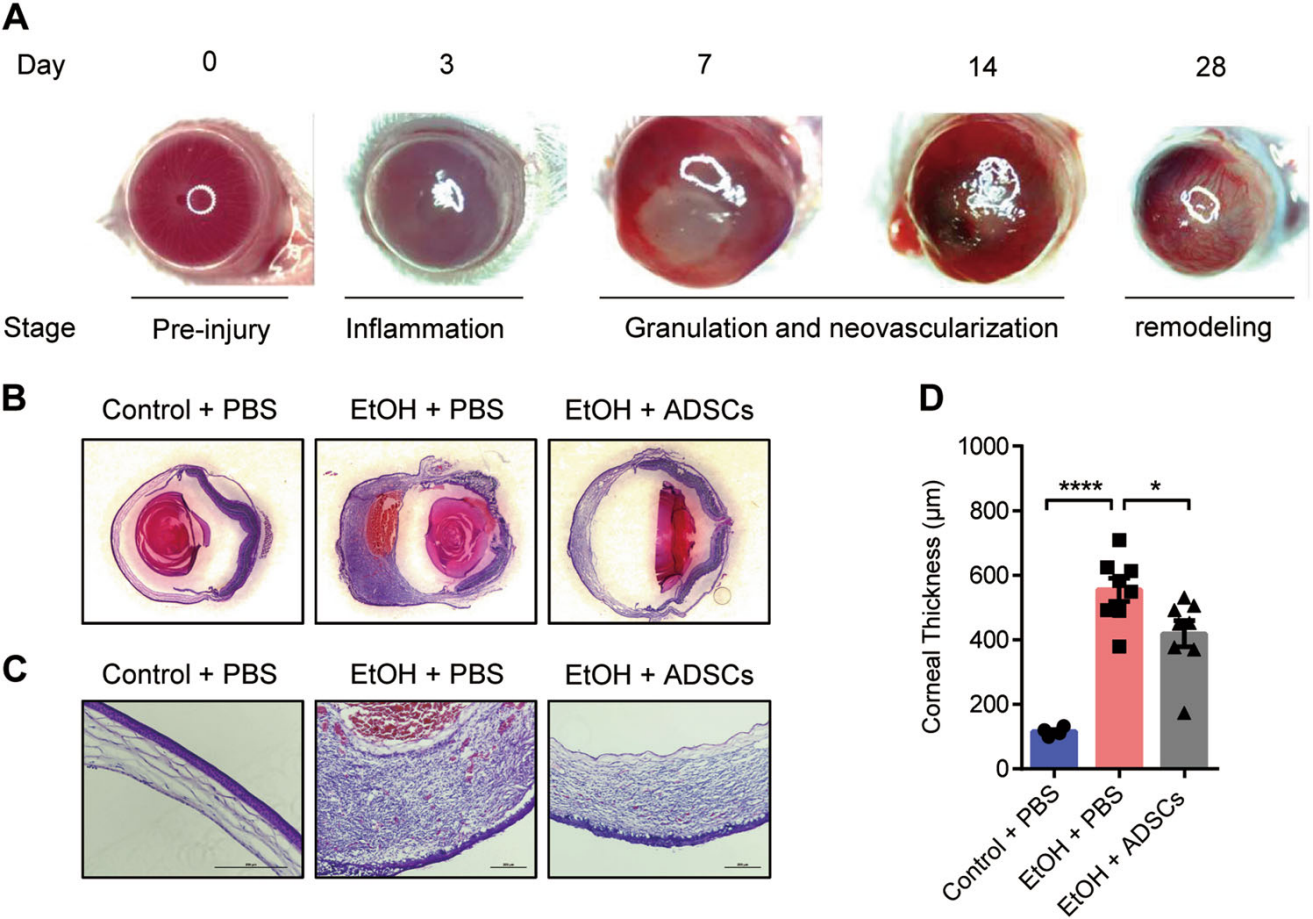
ADSC treatment reduces corneal thickening during granulation. **a** Representative images of the eyeball at different stages of corneal wound healing in the mouse CWH model. **b**, **c** Hematoxylin and eosin staining were performed. The images of whole eyeballs are shown in panel B, and the images of corneas are displayed in panel C (the magnification of the Control + PBS group is 20× and the other two groups are 10×, Scale bar, 200 μm). Mice (*n* = 4–8/group) were treated as described in Fig. 1. The thickness of corneas was measured (**d**). Data are shown as means ± SEM, *P < 0.05; ***P < 0.001 determined by one-way ANOVA with Tukey comparisons.

Various studies have shown that MSCs can promote neovascularization^27–29^. Here, we speculated that ADSCs might indirectly inhibit neovascularization in our CWH model. Examination of the cornea at different stages of the corneal reconstruction process revealed that the corneal stromal tissue was thickened and the anterior segment of the eyeball was enlarged remarkably in the EtOH group during the typical granulation stage (about 7–14 days after injury) (Fig. 2a). The corneal thickness at this stage was significantly reduced in the ADSCs treated group than in the EtOH group (Fig. 2b–d). These data suggest that ADSC treatment can ameliorate corneal granulomatous hyperplasia triggered by EtOH injury.

### ADSC treatment attenuates corneal fibrosis

Granulomatous-like hyperplasia is a characteristic of corneal stromal fibrosis^30^. It has been reported that TGF-β secreted by the corneal epithelial cells can stimulate the corneal fibroblasts (CFs) to differentiate into myofibroblasts in the corneal stroma^31^. It has been shown that corneal myofibroblasts (CMFs) are highly proliferative and can secrete extracellular matrix proteins (ECM) such as collagen, fibronectin, and tenascin C in response to corneal injuries^32^. Since ECM and neovascularization have been implicated in a variety of diseases^33–35^, we wondered if ADSC treatment could reduce the production of ECM. We observed that there was much less collagen deposition after treatment with ADSCs (Supplementary Fig. 2A, B). Furthermore, we examined the expression of genes encoding the ECM components in the cornea and found that the mRNA levels of Col3a1, fibronectin, and tenascin C were significantly decreased after ADSC treatment (Supplementary Fig. 2C). These results suggest that that ADSC therapy may reduce ECM deposition by inhibiting the differentiation of CFs. We therefore examined the expression of α-SMA, a marker of CMFs, in the cornea after ADSC treatment. We found that the expression levels of α-SMA were lower in the EtOH + ADSCs group than in the EtOH group as shown by immunofluorescence staining (Supplementary Fig. 2D). These results strongly suggest that ADSCs inhibit ECM formation of CFs and thus alleviate corneal fibrosis.

### Depletion of peripheral neutrophils during granulation reduces neovascularization

**Fig. 3:**
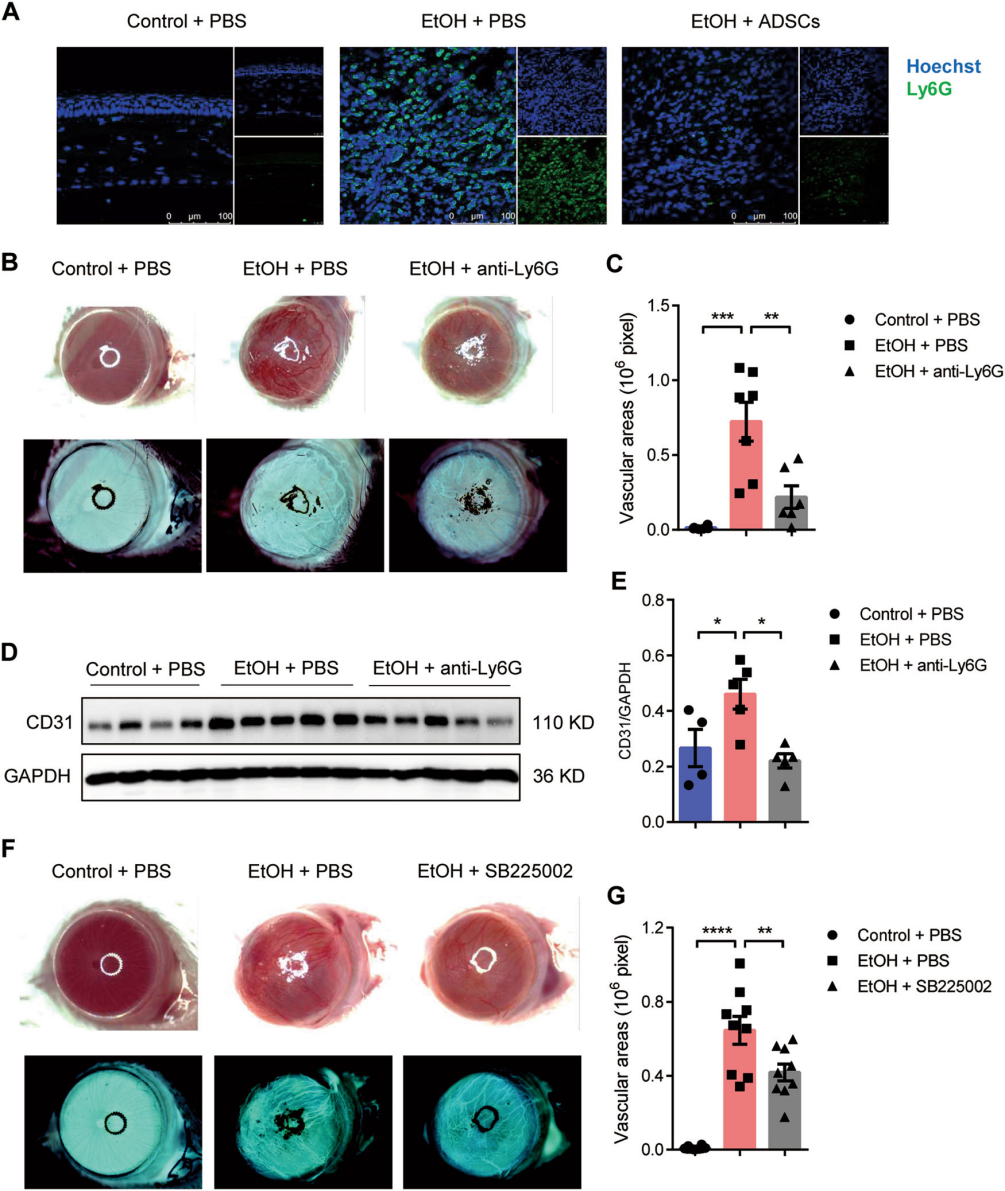
Depletion of peripheral neutrophils during granulation can reduce corneal neovascularization. **a** Mice were treated as described in Fig. 1 and the eyeball samples were collected on Day 7. Anti-Ly6G was used to label neutrophils in corneas, scale bars: 100 μm. **b**–**e** Mice with ethanol exposed eyes were treated with anti-Ly6G or PBS from the 3^rd^ day, and all samples were collected on Day 28. The bright-field photos of eyeballs and their inverted phases (**b**) were taken in each group on Day 21 and the vascular areas of corneas in each group (**c**) were measured (*n* = 6–7 mice/group). The CD31 expression of the whole eyeball in each group was detected by Western blotting analysis (**d**) and the statistics among the groups were carried out (**e**). **f**, **g** Mice with ethanol injured eyes were treated with or without CXCR2 inhibitor SB225002 from the 3^rd^ day, and all samples were collected on Day 28. The bright-field photo of eyeballs and their inverted phases (**f**) were taken in each group on Day 21 and the vascular areas of corneas in each group (**g**) were measured (*n* = 9–10 mice/group). *P < 0.05; **P < 0.01; ***P < 0.001; ****P < 0.0001 determined by one-way ANOVA with Tukey comparisons.

During the granulation stage, we also observed that in the corneal stroma, there were a large number of immune cells, especially Ly6G^+^ neutrophils. As shown in Supplementary Fig. 3A, the number of neutrophils in the eyeball during granulation was about 50 times that of macrophages. Remarkably, after ADSCs treatment, the infiltration of CD11b^+^Ly6G^+^ neutrophil was significantly decreased (Fig. 3a). This prompted us to consider neutrophils as an important player in neovascularization. Therefore, we applied anti-Ly6G antibody every two days, from the day prior the injury induction to the 14^th^ day after injury, to systemically deplete the neutrophils. The depletion effectiveness of peripheral neutrophils was about 98% (Supplementary Fig. 3B). Normally, corneal neovascularization was apparent 21 days after the EtOH treatment, however, in neutrophils depleted mice, EtOH treatment caused severe necrosis of the cornea (Supplementary Fig. 4). This resembles the observation that a large number of neutrophils infiltrated the wound at the early stage during skin wound healing, and neutrophil depletion in the early inflammatory phase delayed the wound healing^36^. This led us to speculate e that neutrophil infiltration in cornea at the early stage of CWH is essential to promote wound healing. In order not to disrupt the early infiltration of neutrophils, we injected anti-Ly6G every two days from the 3^rd^ to 14^th^ day after the injury and found that the neovascularization in the delayed antibody injection group was significantly reduced (Fig. 3b, c). Consistently, the induction of CD31 by EtOH in mouse eyeballs was attenuated (Fig. 3d, e). CXCR2 is a key molecule to mediate neutrophil migration from bone marrow to periphery as inhibition of CXCR2 can reduce the number of peripheral neutrophils^37^. Therefore, we tested whether inhibiting CXCR2 could alleviate corneal neovascularization. We showed that the injection of SB225002, an inhibitor of CXCR2, from the 3^rd^ to 14^th^ day after injury could significantly reduce corneal neovascularization (Fig. 3f, g). These results suggest that neutrophils play an important role in corneal repair and neovascularization. While lack of neutrophils at the initial stage will seriously hamper corneal repair, accelerated clearance of neutrophils during granulation stage could inhibit corneal neovascularization.

### ADSC treatment promotes the clearance of neutrophils at the granulation stage

**Fig. 4:**
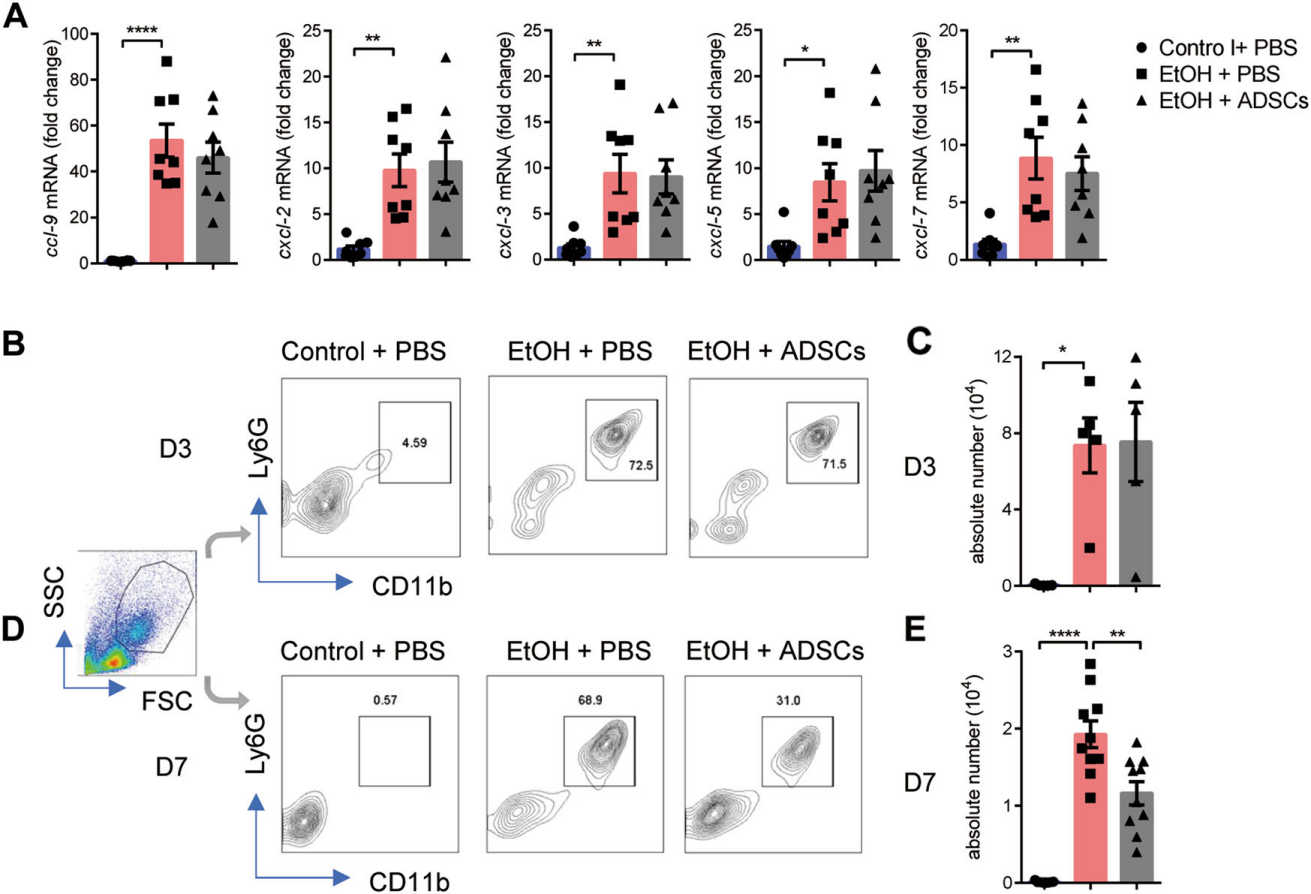
ADSC treatment reduces the excessive retention of neutrophil during the granulation stage. **a**–**e** Mice were treated as described in Fig. 1 and the eyeball samples were collected on Day 3 (**a**–**c**) or Day 7 (**d**, **e**). The mRNA levels of Ccl-9, Cxcl-2, Cxcl-3, Cxcl-5, and Cxcl-7 **a** were determined by qPCR normalized to *β-actin* (*n* = 8 mice/group) on Day 3. **b**–**e** The neutrophils in eyeballs were detected on Day 3 (**b**, **c**) and Day 7 (**d**, **e**), and the percentages (**b**, **d**) and absolute numbers (**d**, **e**) were analyzed by flow cytometry (*n* = 5–10 mice/group). Data are shown as means ± SEM, *P < 0.05; **P < 0.01; ****P < 0.0001 determined by one-way ANOVA with Tukey comparisons.

There are two possible mechanisms to account for the decrease in the number of neutrophils during granulation after ADSCs treatment: reduction in immigration or increase in emigration. We first investigated whether ADSCs could affect neutrophil migration into the eyeball. The mRNA levels of neutrophil chemokines such as Ccl-9, Cxcl-2, Cxcl-3, Cxcl-5, and Cxcl-7 in the eye balls at the inflammatory stage (Day 3) of injury were compared between the EtOH and EtOH + ADSCs groups and found not difference (Fig. 4a). Furthermore, we measured the abundance of neutrophils in the ocular tissue by flow cytometry. We found that the proportion and absolute number of neutrophils at the inflammatory stage were not affected by ADSC treatment (Fig. 4b, c). However, at the granulation stage, the number of neutrophils in the EtOH + ADSCs group was significantly decreased when compared to the EtOH group (Day 7) (Fig. 4d, e), consistent with the result shown in Fig. 4a. These results suggest that while ADSC treatment does not affect the chemotaxis and infiltration of neutrophils at inflammatory stage, it somehow decreases the retention of neutrophils in corneal stroma during granulation.

### ADSCs treatment promotes the emigration of CXCR4^high^ neutrophils

**Fig. 5:**
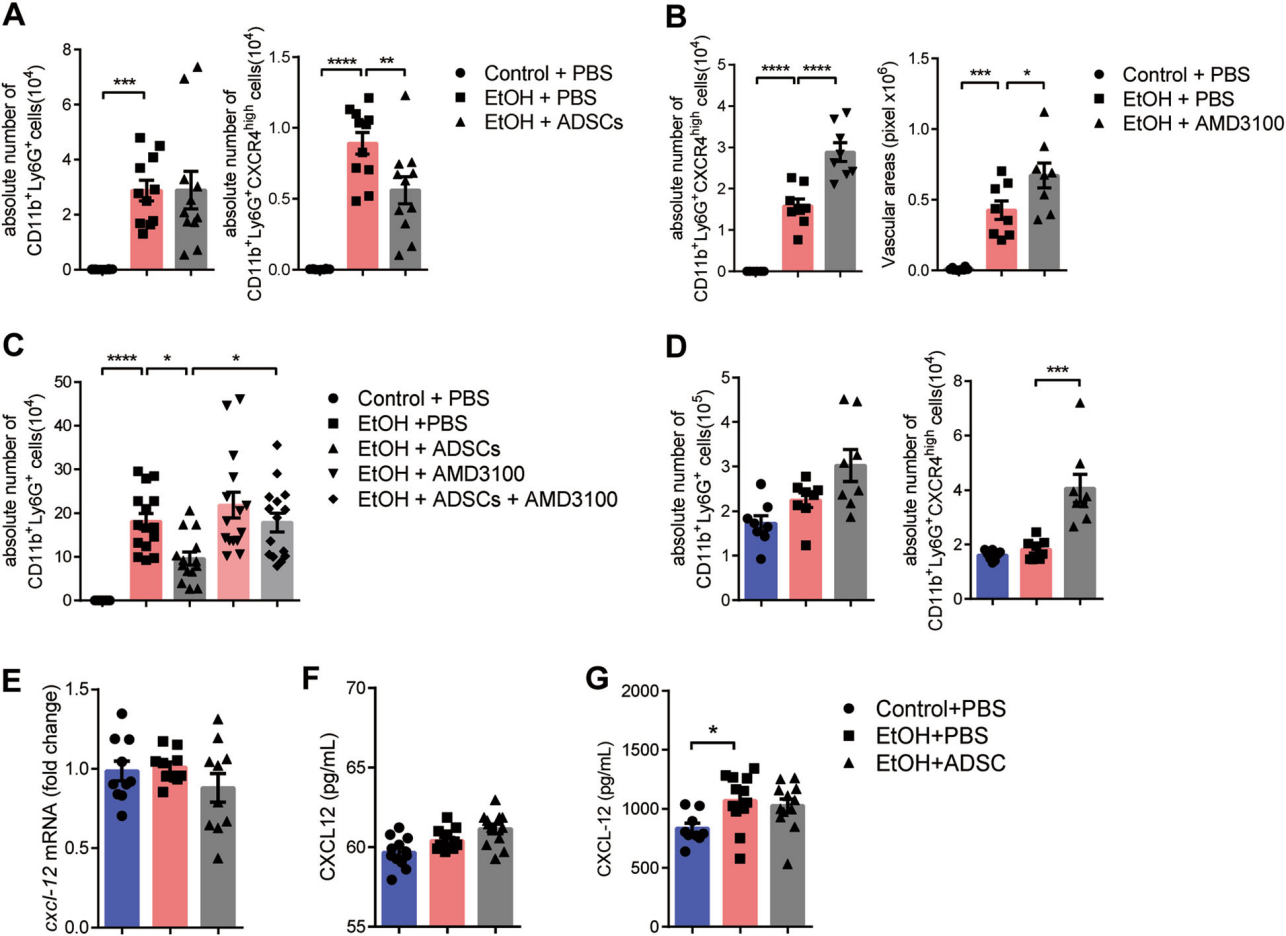
ADSCs treatment promotes the emigration of CXCR4^high^ neutrophils independent of the CXCL12. **a**–**g** Mice were treated as described in Fig. 1. **a** The absolute numbers of total and CXCR4^high^ neutrophils in eyeballs were detected by flow cytometry on Day 3 (*n* = 10–11 mice/group). **b**, **c** Mice with ethanol injured eye were treated with/without AMD3100 and ADSCs, the absolute numbers of total and CXCR4^high^ neutrophils (left **b** and **c**) in eyeballs were detected by flow cytometry on Day 7. And the vascular areas (right **b**) were also measured on Day 7 after AMD3100 treatment (*n* = 8 mice/group in B, *n* = 14–15 mice/group in (**c**). **d** The absolute numbers of total and CXCR4^high^ neutrophils in lungs were detected by flow cytometry on Day 7 (*n* = 8 mice/group). **e** The mRNA levels of Cxcl-12 in the eyeball were determined by qPCR normalized to *β-actin* (*n* = 10 mice/group) on Day 3. **f**, **g** The protein levels of Cxcl-12 in the lung explant supernatant and serum were determined by ELISA (*n* = 12 mice/group) on Day 3. Data are shown as means ± SEM, *P < 0.05; ***P < 0.001; ****P < 0.0001 determined by one-way ANOVA with Tukey comparisons.

Because the chemotaxis of neutrophils at the early phase of injury is not affected by ADSCs, we speculated that the effect of ADSCs could be exerted by promoting the emigration of neutrophils. It has been reported that neutrophils with high expression of CXCR4 migrate to the bone marrow for clearance^37,38^. We found that 3 days after injury, fewer CXCR4^high^ neutrophils were detected in the eyes of the EtOH + ADSCs group than in the EtOH group, though there was no difference in the total numbers of neutrophils between these two groups (Fig. 5a). It has been reported that AMD3100, an inhibitor of CXCR4, can prevent neutrophils from homing to the bone marrow^37,38^. We showed that after the injection of AMD3100, the accumulation of CXCR4^high^ neutrophils were further increased in the eyeball tissues (Fig. 5b). In addition, the injection of AMD3100 can effectively reverse the decreased neutrophil count induced by ADSC treatment (Fig. 5c). Interestingly, the labeled ADSCs were found to migrate into the lungs (Supplementary Fig. 5B). This lung migration pattern of ADSCs corresponded to the reported emigration of neutrophils from injury site to the lung during aseptic liver inflammation^39^. Therefore, we measured the number of neutrophils in the lung tissue in each group on Day 7 and found that while ADSC treatment did not increase the total number of neutrophils, there is a significant increase in CXCR4^high^ neutrophils in the lungs (Fig. 5d). Since the CXCL12/CXCR4 axis was considered to be the key signal pathway to regulate neutrophil homing^37,38^, we tested whether ADSCs affect the retention of CXCR4^high^ neutrophils by regulating the expression of CXCL12 in local or lung tissue. However, examination of *CXCL12* expression in eyeball tissue and lung extract showed no increased expression of CXCL12 by ADSCs (Fig. 5e, f). In addition, there was no significant difference in the concentration of CXCL12 in serum between ETOH group and EtOH + ADSCs group (Fig. 5g). These results suggest that ADSCs treatment could facilitate the emigration of the CXCR4^high^ neutrophils from eye tissue, but it operates independently of the CXCR4/CXCL12 axis.

### ADSCs accelerate neutrophil reverse transmigration by regulating the expression of Jam-c in endothelial cells

**Fig. 6:**
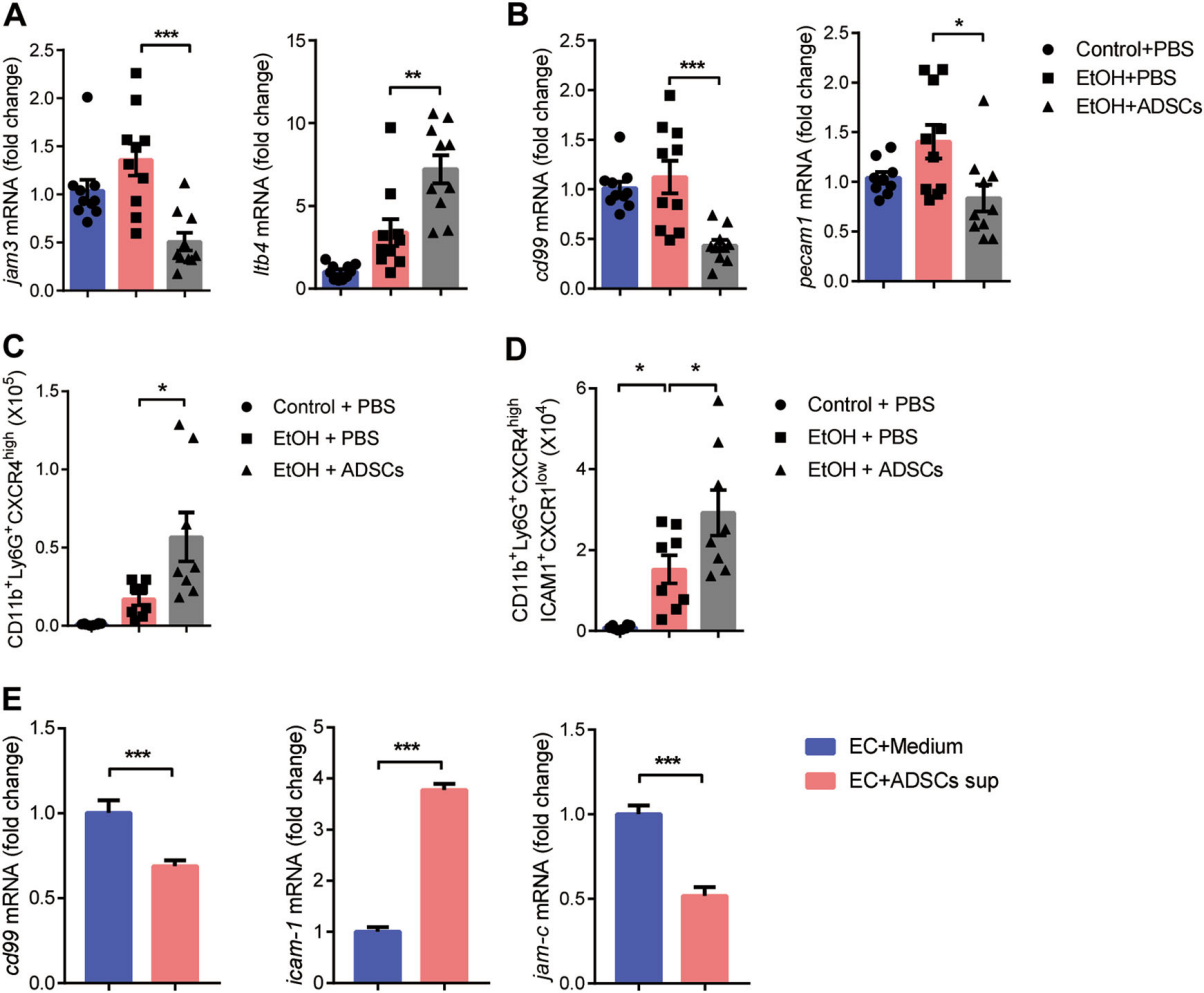
ADSCs accelerate neutrophil reverse transmigration by regulating the expression of Jam-c in endothelial cells. **a**–**d** Mice were treated as described in Fig. 1. **a**, **b** The mRNA levels of Jam-c, Ltb4, CD99, and Pecam1 in the eyeball were determined by qPCR normalized to *β-actin* (*n* = 10 mice/group) on Day 3. **c**, **d** The absolute numbers of CXCR4^high^ neutrophils CXCR4^high^ ICAM-1^+^CXCR1^low^ and in lungs were detected by flow cytometry on Day 7 (*n* = 8 mice/group). **e** The mRNA levels of CD99, ICAM-1 and Jam-c in the HUVEC were determined by qPCR normalized to *β-actin* after co-cultured with medium or ADSCs supernatant. Data are shown as mean ± SEM of four replicates and representative of two independent experiments. *P < 0.05; **P < 0.01; ***P < 0.001 determined by one-way ANOVA with Tukey comparisons or *t* test.

In addition to moving from the vascular lumen to the extravascular tissue (transendothelial cell migration; TEM), neutrophils also exhibit reverse motility through the endothelium (rTEM)^40^. These events were noted in inflammation following ischemia–reperfusion injury, characterized by reduced expression of junctional adhesion molecule C (JAM-C) from EC junctions^41^. Since rTEM can also be one of the reasons for the reduced neutrophil retention in the cornea, we explored whether ADSCs can regulate the rTEM of neutrophils in eye tissue. We found that ADSCs treatment could significantly reduce the expression of Jam-c in eye tissue. Meanwhile, the expression of LTB4 in eye tissue, which has been reported to mediate the reduced expression of endothelial cell JAM-C in I–R model^40^, was up-regulated after ADSCs treatment (Fig. 6a). In addition, EC junctional molecules such as CD99 and Pecam1, which were positively correlated with neutrophil TEM^42–44^, were downregulated after ADSC treatment (Fig. 6b). These results suggest that ADSCs treatment could enhance rTEM of neutrophils in eye tissue without affecting TEM. Since there was more accumulation of CXCR4^high^ neutrophils in the lungs in ADSCs group, we next tested whether these neutrophils may have translocated via rTEM. Studies have shown that neutrophils that have undergone rTEM can be distinguished by high expression of ICAM-1 and low expression of CXCR1, which serve as useful markers for identifying rTEM neutrophils^41,45,46^. We observed that the absolute number of ICAM1^+^CXCR1^low^ neutrophils in CXCR4^high^ neutrophils was significantly higher in the lung tissue after ADSC treatment (Fig. 6c, d). Since DiR-labeled ADSCs could still be detected around the eyeball 14 days after injection (Supplementary Fig. 5A), and Jam-c is mainly expressed in endothelial cells^41,47^ we wonder whether ADSCs can directly regulate the expression of Jam-c in endothelial cells. We found that the expression of Jam-c in endothelial cells was significantly decreased by ADSC culture supernatant, which was consistent with the results in eye tissues (Fig. 6e). Furthermore, ADSC culture supernatant could down-regulate the expression of CD99 and significantly upregulate the expression of adhesion molecule Icam-1 on endothelial cells (Fig. 6e). These results suggest that ADSCs can promote the rTEM of neutrophils in cornea by regulating the expression of Jam-c in vascular endothelial cells, thus reducing the retention of CXCR4^high^ neutrophils.

### NETs regulate corneal neovascularization

**Fig. 7:**
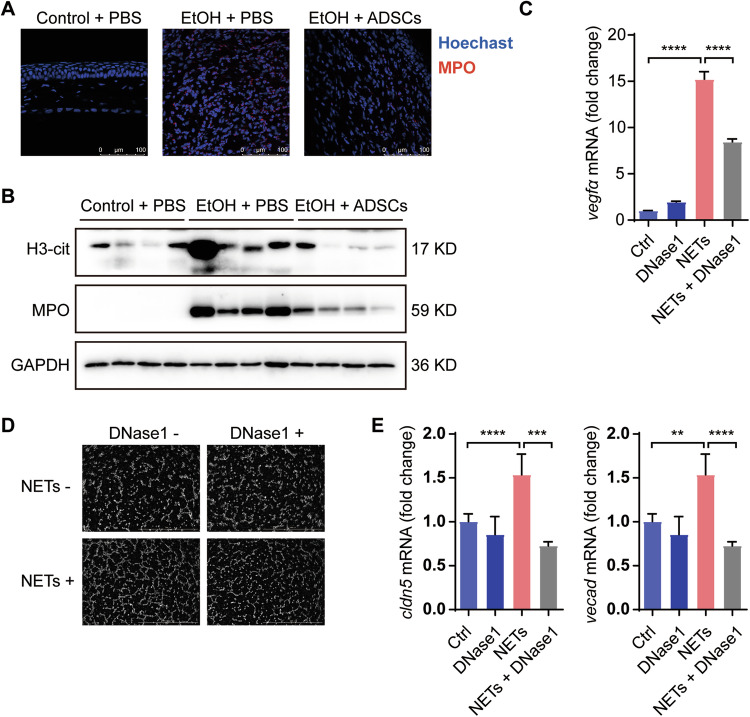
Neutrophil extracellulartraps (NETs) promote corneal neovascularization. **a**, **b** Mice were treated as described in Fig. 1. Corneas in each group were collectedon Day 7 and stained for MPO (**a**), scale bars: 100 μm, and the protein levels of H3-cit and MPO were detected by Western blot (**b**). After treatment with NETs and/or DNase1 (100 ug/mL) for 24 h, CFs were analyzed for Vegfa mRNA level (**c**). Tube-formation abilities of EAhy926 cells were detected after NETs and/or DNase1 (100 μg/mL) treatment (**d**), scale bars: 1 mm. The mRNA levels of Cldn5 and Vecad in EAhy926 cells were detected 3 h after NETs and/or DNase1 (100 μg/mL) treatment (**e**). Data are shown as mean ± SEM of four (**c**, **e**) replicates and representative of two independent experiments. Data are shown as means ± SEM, *P < 0.05; **P < 0.01; ***P < 0.001; ****P < 0.0001 determined by one-way ANOVA with Tukey comparisons.

It has been reported that neutrophils can form NETs in aseptic inflammatory tissues^48^. We also found in our cornea injury model that the expression of MPO, a marker of NETs, was significantly increased in cornea stroma on the 7^th^ day after injury. Importantly, ADSC treatment dramatically lowered MPO expression (Fig. 7a). The expression of H3-cit and MPO in the whole eyeball was also elevated (Fig. 7b). We subsequently tested whether the NETs contribute to neovascularization during the repair of corneal injury. We induced NETs in vitro and then co-cultured them with CFs. NETs were found to upregulate the expression of Vegfa in CFs. This induction of Vegfa was partially blocked by DNase1 treatment (Fig. 7c). In an assay using EAhy926 human endothelial cells, we showed that NETs increased neovascularization and DNase I significantly reduced it (Fig. 7d). This neovascularization-promoting effect of NETs is also supported by the increased expression of Cldn5 and Vecad in endothelial cells (Fig. 7e).

Taken together, our studies revealed that neutrophils at the granulation stage can form NETs locally. These NETs then directly promote neovascularization. Administration of ADSCs reduces neutrophil retention in the cornea stroma and thus minimizing the NET formation and corneal vascularization.

## Discussion

Cornea are devoid of any blood vessels. However, when corneal injury occurs, vasculatures are formed to transport nutrients and immune cells as an essential part of the repair and regeneration cascade. The reparation often results in either complete restoration of the clear cornea or the formation of persistent blood vessels that cloud the cornea. Therefore, the reduction in corneal neovascularization has always been a challenge in ophthalmology. Based on their pro-angiogenic roles in the tumors and other wounded tissues, VEGF, PDGF, and MMP family members have been explored as candidate targets for keeping the transparency of the cornea^49–51^. Mesenchymal stem/stromal cells were also explored as a therapeutic for corneal neovascularization^13^. This effect of MSCs was partially due to TSG-6, as TSG-6 was shown to be an effector in this application^52^. However, because the corneal repair process was not monitored for all stages in the previous studies, how mesenchymal stem cells participate in the entire repair process remains largely unknown.

In this study, to better simulate clinical situations, we followed the corneal repair process for an extended time, longer than 3 weeks, and further investigated the mechanisms underlying the ameliorating effect of adipose MSCs on corneal repair. We verified that ADSCs could inhibit corneal neovascularization (Fig. 1). Compared to the short-term follow-up used in previous studies, the long-term follow-up (more than 3 weeks) has two important advantages. First, after three weeks, the corneal repair process has entered the remodeling stage and the shape and size of the cornea are basically restored to their original dimensions, enabling a more accurate evaluation of neovascularization. Second, the extended evaluation allowed us to examine the different stages along a longer repairing process. We found that the most noticeable event in the repairing process is granulation, which is typically accompanied by fibrosis. Corneal fibrosis, also known as collagen fiber recover and scar formation, is characterized by myofibroblast differentiation and proliferation, corneal stromal thickening and the production of a large amount of extracellular matrix (ECM). The ECM is not only one of the main structural components in various tissues, but also plays a vital role in neovascularization during physiological and pathological conditions^33^. It provides a platform for angiogenic signaling by increasing the bioavailability of soluble angiogenic factors, and by enhancing the interaction between ECM components and their cellular transmembrane receptors^34,35^. We found that after ADSC treatment, the expression of α-SMA was significantly decreased in the eyes, and the ECM components, such as collagen, fibronectin, and tenascin C, were also decreased. A previous study showed that aged fibroblasts overexpressing ECM components are associated with corneal neovascularization^8^. Therefore, we concluded that ADSCs could reduce neovascularization by inhibiting corneal fibrosis.

The other feature that accompanied granulation is the persistent infiltration of neutrophils in the corneal stroma. Unlike skin injury-induced rapid mobilization of neutrophils (within 30 min) and the subsequent replacement by macrophages (2–3 days), neutrophil infiltration to the damaged cornea can be seen throughout the inflammatory and granulation stages. We found that on the 7^th^ day after injury, the number of neutrophils in the eyeball was still about 50 times that of macrophages (Supplementary Fig. 3A). This result led us to believe that neutrophils, which are overwhelmingly dominant in numbers, are very important for CWH. By depleting neutrophils starting at different time points during CWH, we showed that neutrophil infiltration in the early stage is necessary and beneficial for corneal repair (Supplementary Fig. 4). However, depleting neutrophils after significant infiltration reduced neovascularization afterwards, indicating that long-term retention of neutrophils in the eyeball is critical for promoting neovascularization (Fig. 3b, c). While ADSC treatment had little effect on the early neutrophil infiltration, it significantly reduced the number of neutrophils at the granulation stage (Fig. 4). This suggests that that ADSCs may regulate the retention of neutrophils at the injury site.

It has been unequivocally demonstrated that neutrophils may emigrate through the endothelium^40,41^. Senescent neutrophils were shown to migrate to bone marrow for clearance, a process dependent on the increased CXCR4 expression on those aged neutrophils that allows their homing to bone marrow via CXCL12-mediated chemotaxis^37,38^. In the corneal injury model, we found that 3 days after injury, there were also fewer CXCR4^high^ neutrophils in the eyes of the EtOH + ADSCs group than in those of the EtOH group (the two groups had similar neutrophil counts in the eyeball at this time) (Fig. 5a). This result suggests that the ADSC treatment may have increased the emigration of aged neutrophils. Since a previous study showed that neutrophils during liver aseptic inflammation migrate from the injury site to the lungs^39^, we examined neutrophils in the lungs as well and found that except neutrophils, ADSCs were also found in the lungs (Fig. 5d). Therefore, we speculated that ADSCs may function to promote the mobilization of CXCR4^high^ neutrophils to the lungs. Indeed, the number of CXCR4^high^ neutrophils in the lungs of ADSC treated group was significantly higher than that in the EtOH group. Importantly, upon the application of CXCR4 inhibitor, AMD3100, CXCR4^high^ neutrophils in the cornea increased and resulted in more severe neovascularization (Fig. 5b, c). These results suggest that the emigration of aged neutrophils is a significant factor in the regulation of neovascularization, and ADSCs can reduce corneal neovascularization by promoting the migration of CXCR4^high^ neutrophils.

Although many studies suggest that the CXCR4^high^ neutrophils are regulated by CXCL12^53–55^, in our model, we did not find that EtOH + ADSCs group, which aggregates more CXCR4^high^ neutrophils in the lung, showed neither increased CXCL12 expression in the lung nor decreased CXCL12 expression in the eyeball (Fig. 5e, f). These results suggest that ADSCs did not mediate neutrophil emigration by affecting chemotactic signals. On the other hand, neutrophils can also exhibit motility away from sites of inflammation and injury, which known as the reverse migration of neutrophils.The enhancement of rTEM, which is positively correlated with the low expression of adhesion molecule Jam-C in endothelial cells, can reduce the retention of neutrophils in tissues. We found that ADSC treatment can not only increase the number of rTEM neutrophils in the lung, but also effectively reduce the expression of Jam-c in eye tissue (Fig. 6a–d). Co-culture of ADSCs and endothelial cells can also reduce the expression of Jam-C in endothelial cells (Fig. 6e). Although the specific factor(s) produced by ADSCs has yet to be identified, these results suggest that ADSCs can enhance the rTEM of neutrophils by regulating the expression of Jam-c in endothelial cells, thus reducing the retention of neutrophils in corneal.

Neutrophils have been reported to promote neovascularization in a variety of ways^56–58^, one of which is through the NETs^26^. NETs are structures released from neurtropils composed of chromatin filaments coated with histones, proteases, and granular and cytosolic proteins, which are believed to allow neutrophils to catch and kill bacteria. However, increasing evidence suggests that this process also occurs in sterile inflammation^48^. We found that the expression of two NETs markers, MPO and H3-cit, was upregulated in the cornea during granulation (Fig. 7b). Therefore, it is possible that the neutrophils that fail to emigrate to the bone marrow or lungs promptly may form NETs in the corneal stroma and promote neovascularization. Our experiments indeed showed that DNase1 could reduce tube formation of endothelial induced by NETs (Fig. 7d). Nevertheless, this important role of neutrophil retention may be manifold and need to be further explored.

**Fig. 8:**
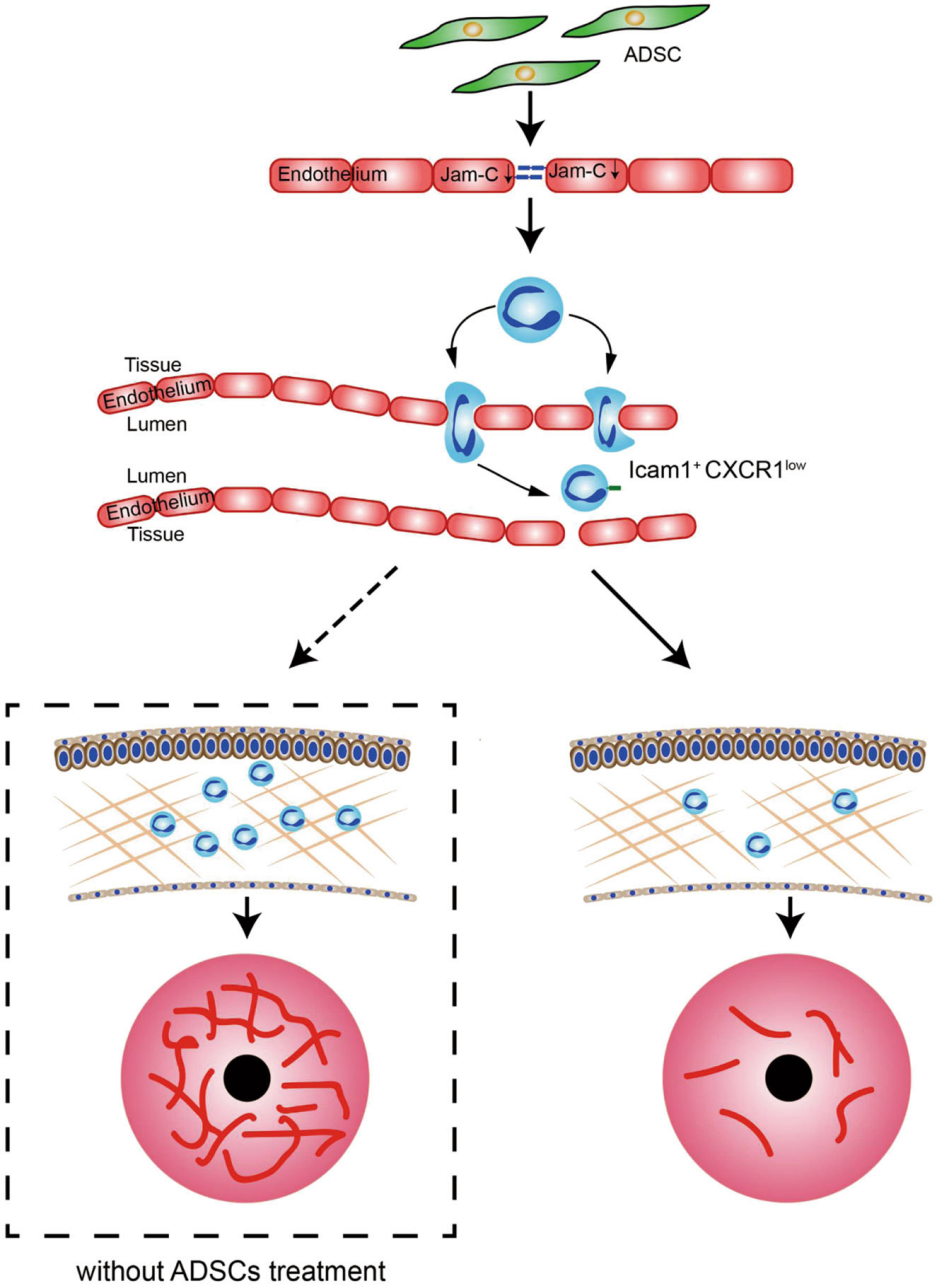
A diagrammatic model of the role of neutrophils in corneal neovascularization and its modulation by ADSC. Prolonged retention of neutrophils promotes corneal neovascularization. ADSCs promote the reverse migration of neutrophils from the corneal tissue to the blood vessels. Due to the enhanced reverse migration of neutrophils, the retention of neutrophils in corneal stroma was reduced, which attenuates neovascularization in cornea.

In summary, our work revealed that ADSCs can effectively inhibit neovascularization caused by corneal injury. Importantly, we found that neutrophils are critically involved in corneal wound healing and have opposite effects at different stages of the healing process. We demonstrated that ADSCs can attenuate injury-induced neovascularization, possibly by enabling the prompt clearance of neutrophils from injury sites. Our results suggest that the kinetics of neutrophil migration is a accurately regulated process during corneal wound healing, and complete absence and excessive retention of neutrophils can both impair or derail the desirable healing process (Fig. 8).

## Supplementary information

Supplement Figure 1

Supplement Figure 2

Supplement Figure 3

Supplement Figure 4

Supplement Figure 5

Supplement Figure Legend

Supplement Table 1

Supplement Table 2
