# Correction: Adipose-derived mesenchymal stromal cells promote corneal wound healing by accelerating the clearance of neutrophils in cornea

**DOI:** 10.1038/s41419-024-07295-0

**Published:** 2025-01-21

**Authors:** Qianwen Shang, Yunpeng Chu, Yanan Li, Yuyi Han, Daojiang Yu, Rui Liu, Zhiyuan Zheng, Lin Song, Jiankai Fang, Xiaolei Li, Lijuan Cao, Zheng Gong, Liying Zhang, Yongjing Chen, Ying Wang, Changshun Shao, Yufang Shi

**Affiliations:** 1 The First Affiliated Hospital of Soochow University, State Key Laboratory of Radiation Medicine and Protection, Institutes for Translational Medicine, Soochow University Medical College, Suzhou, Jiangsu 215123 China; 2 Department of Ophthalmology, The Affiliated Hospital of Jiangnan University, 200 Huihe Road, Wuxi, 214062 China; 3 The Second Affiliated Hospital of Soochow University, Suzhou, Jiangsu 215123 China; 4 Key Laboratory of Stem Cell Biology, Shanghai Jiao Tong University School of Medicine and Shanghai Institutes for Biological Science, Chinese Academy of Sciences, Shanghai, 200025 China

Correction to: *Cell Death and Disease* 10.1038/s41419-020-02914-y, published online 26 August 2020

We wish to correct an error involving an inadvertently misplaced image in one of the figure panels. In Figure 7D, the top two images were meant to represent two distinct negative control groups: one with DNase1 and one without (both in the absence of NETS). However, both images were mistakenly taken from the group treated with DNase1. The correction does not affect the conclusion of the article. The authors apologize for the error, and remain committed to maintaining the highest standards of scientific accuracy and integrity in our research and publications.
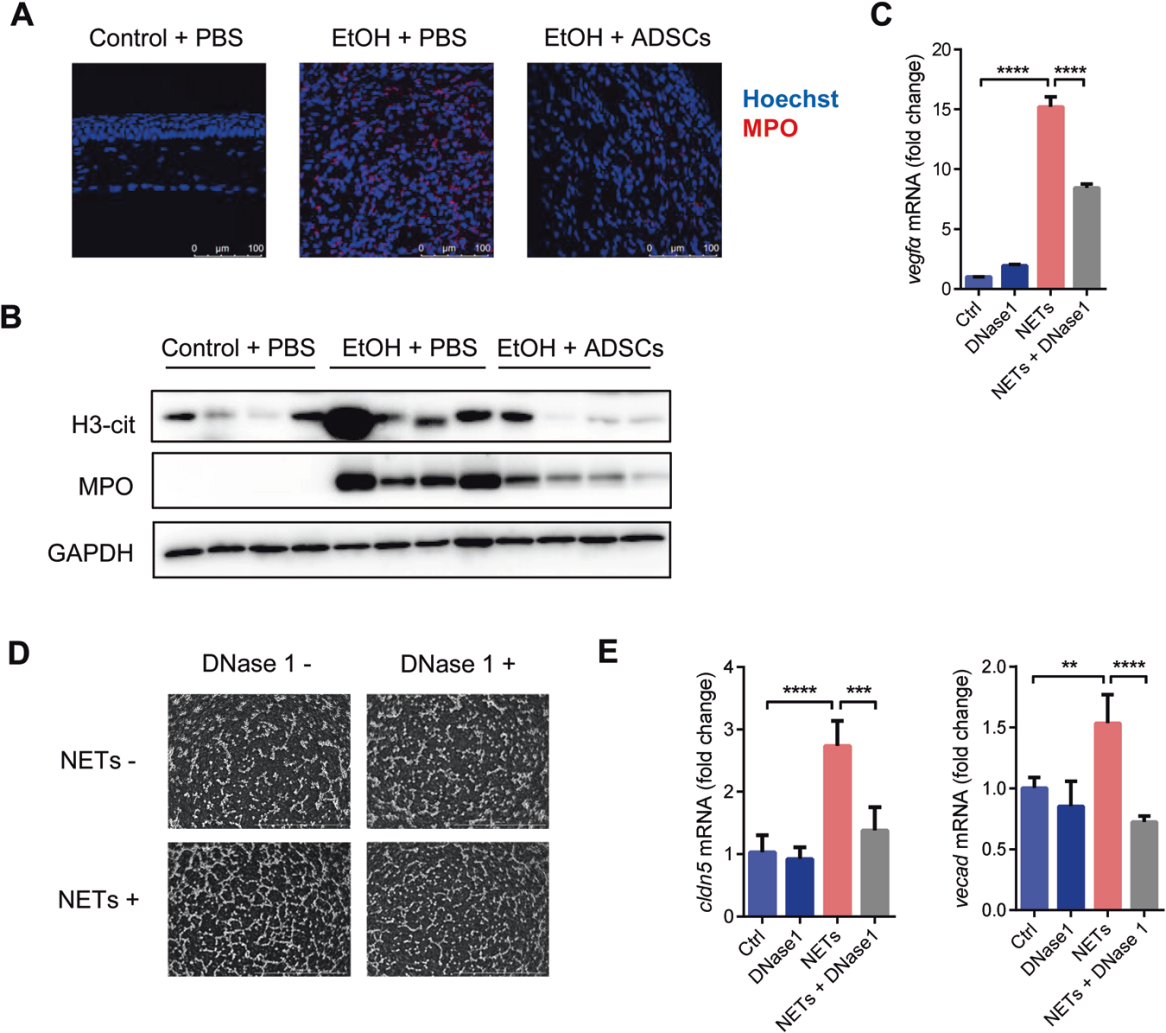


**Figure 7 Neutrophil extracellular traps (NETs) promote corneal neovascularization.** **A**, **B** Mice were treated as described in Fig. 1. Corneas in each group were collected on Day 7 and stained for MPO (**A**), scale bars: 100 μm, and the protein levels of H3-cit and MPO were detected by Western blot (**B**). After treatment with NETs and/or DNase1 (100 μg/mL) for 24 h, CFs were analyzed for Vegfa mRNA level (**C**). Tube-formation abilities of EAhy926 cells were detected after NETs and/or DNase1 (100 μg/mL) treatment (**D**), scale bars: 1 mm. The mRNA levels of Cldn5 and Vecad in EAhy926 cells were detected 3 h after NETs and/or DNase1 (100 μg/mL) treatment (**E**). Data are shown as mean ± SEM of four (**C**, **E**) replicates and representative of two independent experiments. Data are shown as means ± SEM, *P < 0.05; **P < 0.01; ***P < 0.001; ****P < 0.0001 determined by one-way ANOVA with Tukey comparisons.

The original article has been corrected.

