# Supplementary figures and images for: Adipose-derived mesenchymal stromal cells promote corneal wound healing by accelerating the clearance of neutrophils in cornea
Source: Cell Death Dis. 2020 Aug 26;11(8):707. doi: 10.1038/s41419-020-02914-y (PMC7450061; doi:10.1038/s41419-020-02914-y)

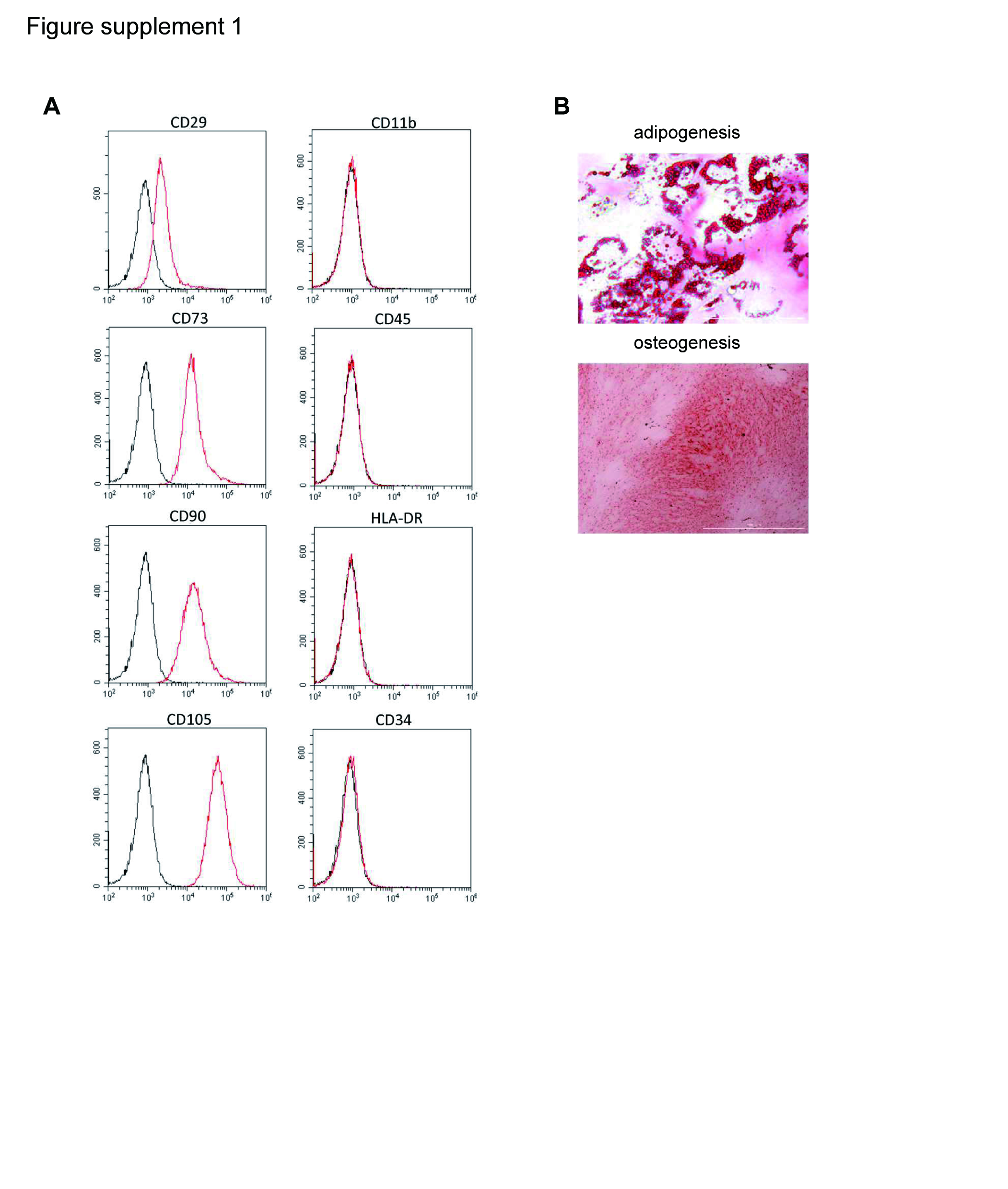

Supplement: Supplementary file 1 — Supplement Figure 1 [file 41419_2020_2914_MOESM1_ESM.tif]

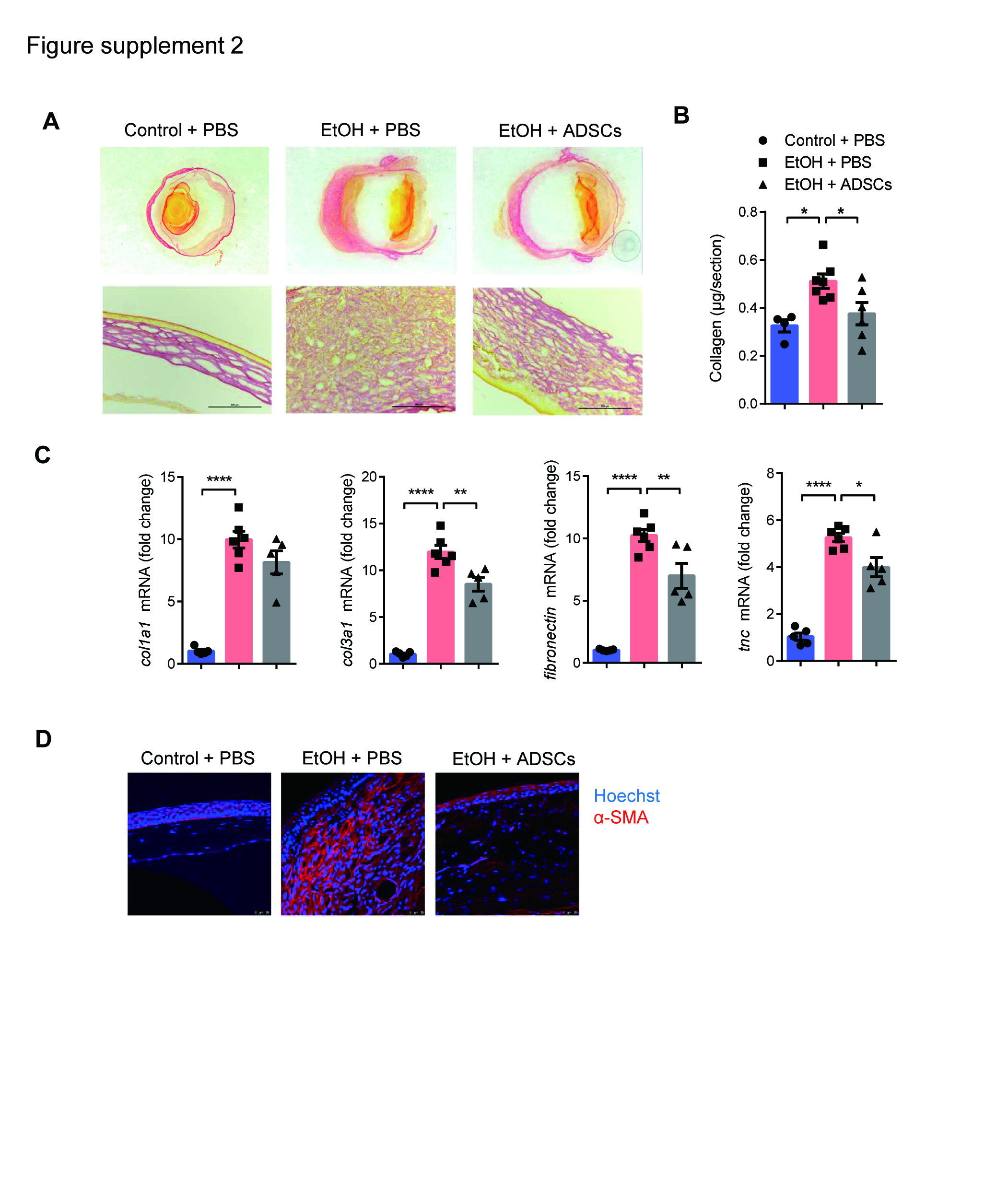

Supplement: Supplementary file 2 — Supplement Figure 2 [file 41419_2020_2914_MOESM2_ESM.tif]

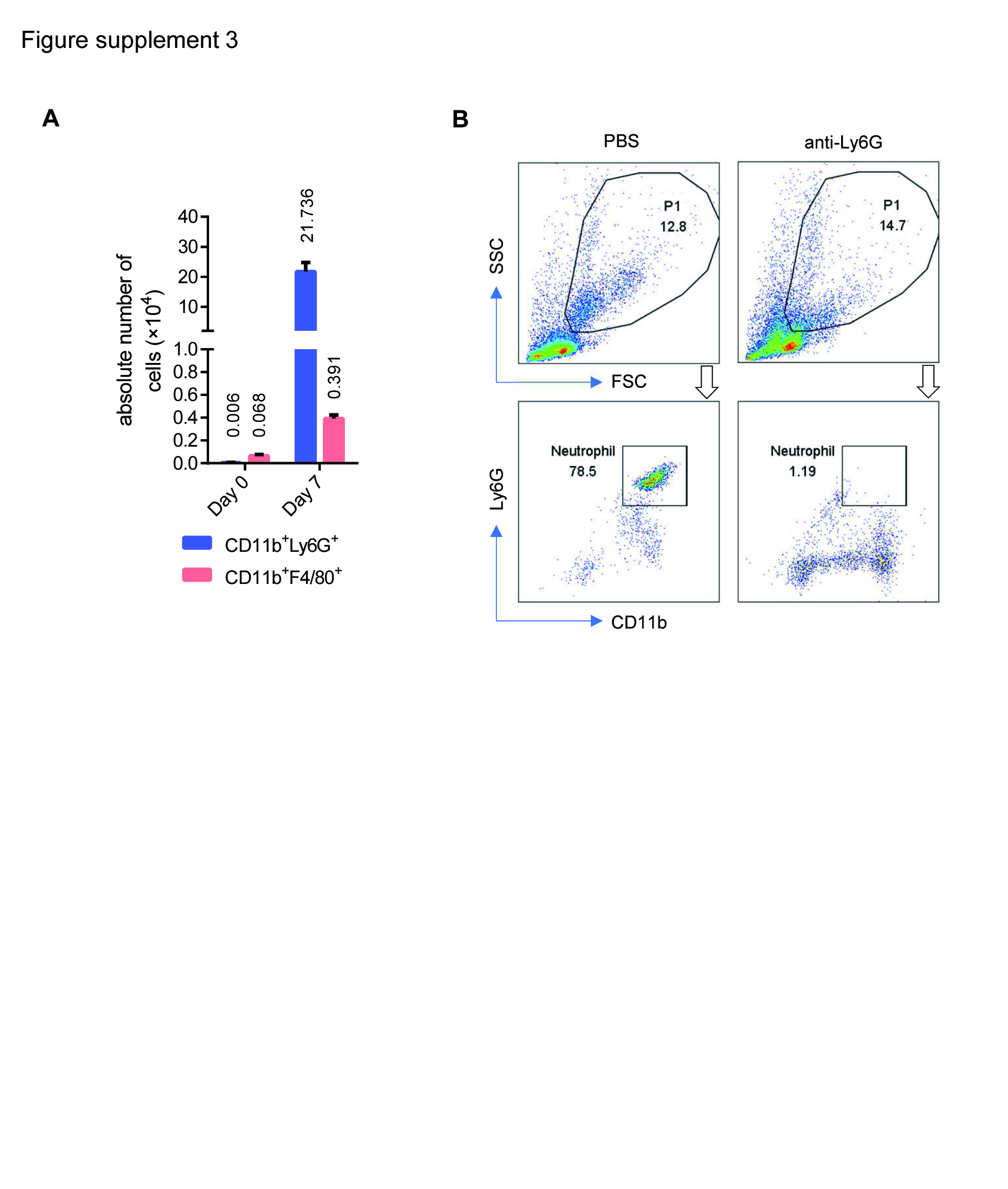

Supplement: Supplementary file 3 — Supplement Figure 3 [file 41419_2020_2914_MOESM3_ESM.tif]

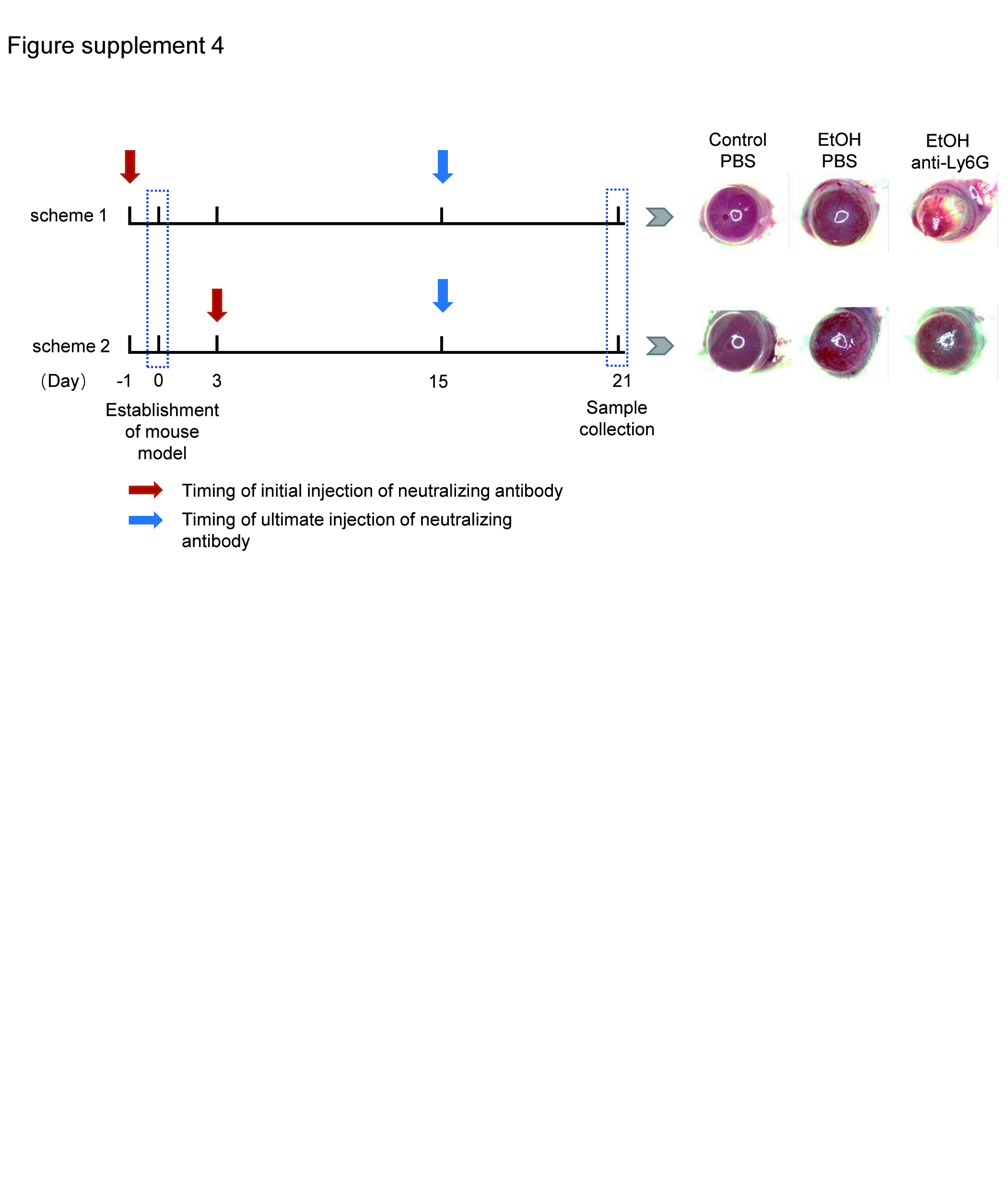

Supplement: Supplementary file 4 — Supplement Figure 4 [file 41419_2020_2914_MOESM4_ESM.tif]

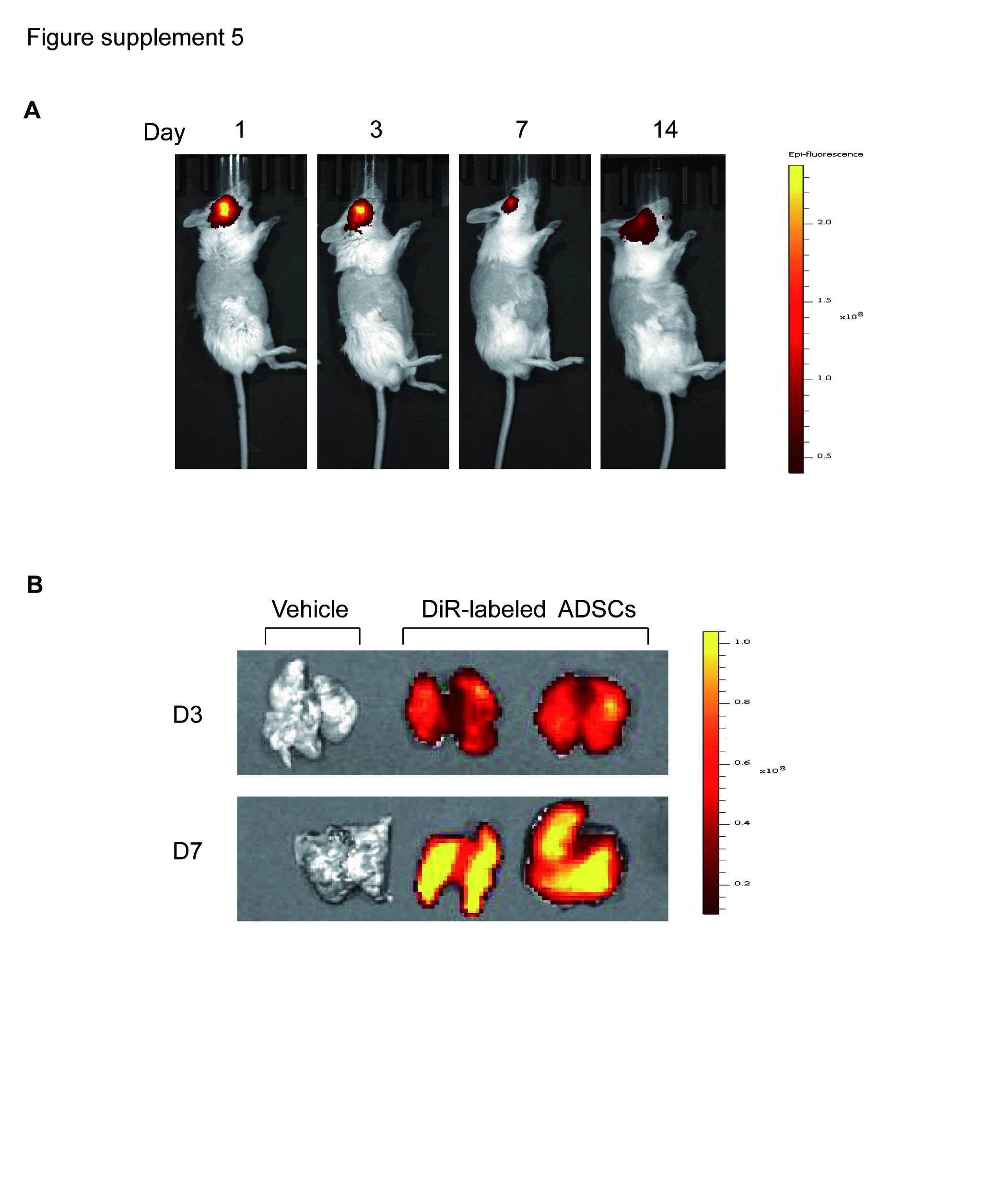

Supplement: Supplementary file 5 — Supplement Figure 5 [file 41419_2020_2914_MOESM5_ESM.tif]
