# Supplementary material for: Adipose-derived mesenchymal stromal cells promote corneal wound healing by accelerating the clearance of neutrophils in cornea
Source: Cell Death Dis. 2020 Aug 26;11(8):707. doi: 10.1038/s41419-020-02914-y (PMC7450061; doi:10.1038/s41419-020-02914-y)
Supplement: Supplementary file 6 — Supplement Figure Legend [file 41419_2020_2914_MOESM6_ESM.docx]

**Figure supplement 1. Characterization of human adipose derived mesenchymal stromal cells.**

(A) Flow cytometry was employed to identify the major markers of hADSCs, with primary antibody as indicated. (B) The adipogenesis and osteogenesis of ADSCs in vitro，Scale bars represent 1 mm.

**Figure supplement 2. ADSC treatment attenuates corneal fibrosis**

(A-E) Mice were treated as described in Fig.1 and the eyeball samples were collected on day 7. Sirius Red Stain of eyeballs slices were performed (A) and the amount of collagen in slices (*n* = 4–7 mice/group) were measured (B) scale bar, 200μm. The mRNA levels of *col1a1, col3a1, fibronectin,* and *tnc* (C) were determined by quantitative PCR (qPCR) normalized to GAPDH (*n* = 5-6 mice/group). The expression of α-SMA of corneal were (scale bars: 25 μm) detected by IF (D). *P < 0.05; **P <0.01; ***P <0.001; ****P < 0.0001 determined by one-way ANOVA with Tukey comparisons.

**Figure supplement 3. Anti-Ly6G can effectively deplete neutrophils in peripheral blood.**

(A) The absolute number of macrophages（CD11b^+^F4/80^+^）and neutrophils (CD11b^+^/Ly6G^+^) in the eyeballs on day 0 and day 7 after EtOH injury. The number represents the mean of each column (n = 8 mice/group). (B) Male BALB/c mice were injected intraperitoneally with anti-Ly6G or PBS. Peripheral blood samples were taken 24 hours later. After lysing red blood cells, antibodies specific for CD11b and Ly6G were used to label neutrophils. Flow cytometry was used to detect the proportion of double positive cells. The result shows that the depletion effectiveness is about 98%.

**Figure supplement 4. Two intervention schemes with anti-ly6G and their respective effects on corneal neovascularization.**

In scheme 1, antibody injection was started from the day before cornea injury induction to the 14th day after molding. There was severe necrosis 7 days after the last treatment; In scheme 2, antibody injection was started from the third day to the 14th day after modeling. Corneal neovascularization in the antibody injection group was reduced when compared to the IgG group after 21 days.

**Figure supplement 5. Retro-orbital injection of ADSCs remained at the injection site for a prolonged period of time.**

(A) *In vivo* imaging of DiR-labeled ADSCs at indicated time-points following retro-orbital injection. (B) DiR-labeled ADSCs or PBS were injected into the retro-orbital of mice with ethanol injured eye. Three and seven days later, the lung localization of ADSCs were detected for fluorescence imaging.
