# Supplementary material for: Adipose-derived mesenchymal stromal cells promote corneal wound healing by accelerating the clearance of neutrophils in cornea
Source: Cell Death Dis. 2020 Aug 26;11(8):707. doi: 10.1038/s41419-020-02914-y (PMC7450061; doi:10.1038/s41419-020-02914-y)
Supplement: Supplementary file 7 — Supplement Table 1 [file 41419_2020_2914_MOESM7_ESM.docx]

**Table.1. Reagents**

| Reagent type (species)  or resource | Designation | Source or  reference | Identifiers | Additional  information |
| --- | --- | --- | --- | --- |
| Antibody | Anti-CD31  (rabbit polyclonal) | Abcam | Cat# ab28364 | 1:50 IF  1:500 WB |
| Antibody | Anti-α-smooth muscle actin  (rabbit polyclonal) | Abcam | Cat# ab5694 | 1:100 IF  1:500 WB |
| Antibody | Anti-Ly6G  (rat monoclonal) | Abcam | Cat# ab25377 | 1:300 IF |
| Antibody | Anti- myeloperoxidase  (rabbit monoclonal) | Abcam | Cat# ab208670 | 1:100 IF  1:1000 WB |
| Antibody | Anti- Histone H3 (citrulline R2 + R8 + R17)  (rabbit polyclonal) | Abcam | Cat# ab5103 | 1:1000 WB |
| Antibody | Anti-Neutrophil Elastase antibody | Abcam | Cat# ab68672 | 1:1000 WB |
| Antibody | Anti-GAPDH  (rabbit monoclonal) | CST | Cat# 5174S | 1:5000 WB |
| Antibody | Alexa 488-conjugated-goat anti-rabbit IgG | Abcam | Cat# ab150077 | 1:500 |
| Antibody | Alexa 594-conjugated-goat anti-rat IgG | Abcam | Cat# ab150160 | 1:500 |
| Antibody | Alexa 555-conjugated-goat anti-mouse IgG | Thermo Fisher Scientific | Cat# A31570 | 1:500 |
| Antibody | CD11b Efluor 450 | eBioscience |  | Mouse  1:300 |
| Antibody | Sca1 PE | eBioscience |  | Mouse  1:300 |
| Antibody | Gr1 Al700 | eBioscience |  | Mouse  1:300 |
| Antibody | CD11b APC | eBioscience |  | Mouse  1:300 |
| Antibody | CXCR4 PE | eBioscience |  | Mouse  1:300 |
| Antibody | CD181 (CXCR1) PE | BD Pharmingen | 566383 | Mouse  1:300 |
| Antibody | Ly6G | eBioscience |  | Mouse  1:300 |
| Antibody | CD29 PE | eBioscience |  | Human  1:300 |
| Antibody | CD11b PE  Colne : | eBioscience |  | Human  1:300 |
| Antibody | CD73 PE | eBioscience |  | Human  1:300 |
| Antibody | CD45 PE | eBioscience |  | Human  1:300 |
| Antibody | HLA-DR PE | eBioscience |  | Human  1:300 |
| Antibody | CD105 PE | eBioscience |  | Human  1:300 |
| Antibody | CD90 PE | eBioscience |  | Human  1:300 |
| Antibody | CD34 PE | eBioscience |  | Human  1:300 |
| Antibody | Anti-mouse Ly-6G  Clone:1A8 | Biolegend | Cat# 127649 |  |
| Chemical compound, drug | SB225002 | Selleck | Cat# S7651 |  |
| Chemical compound, drug | AMD3100 | Selleck | Cat# S8030 |  |
| Enzyme | Collagenase Ⅰ | Thermo Fisher Scientific | Cat# 17100017 |  |
| Enzyme | Collagenase II | Thermo Fisher Scientific | Cat#17101015, |  |
| Enzyme | DISPASE | Thermo Fisher Scientific | Cat# 17105041 |  |
| Enzyme | DNase I | solarbio | Cat# D8071 |  |
| Material | Matrigel | BD Pharmingen | Cat# 356234 |  |
| Chemical compound | DiR | PerkinElmer | 125964 |  |
| Kit | SYBR SELECT MASTER MIX | Thermo Fisher Scientific | Cat# 4472920 |  |
| Kit | Total RNA extracted kit | Fastagen | Cat# RNA fast200 |  |
| Kit | Pierce BCA protein assay kit | Thermo Fisher Scientific | Cat# 23227 |  |
| Kit | Mouse Neutrophil Negative Selection Kit | STEM CELL | Cat# 19762 |  |
| Kit | Picro-Sirius Red Stain Kit  (For Collagen) | ScyTek | Cat# PSR-1 |  |
| Kit | Sirius Red/Fast Green Collagen Staining Kit | Chondrex | Cat# 9046 |  |
| Kit | LEGEND MAX™ Mouse CXCL12 (SDF-1β) | BioLegend | Cat# 444207 |  |
| Medium | Endothelial Cell Growth Medium | PromoCell | Cat# C-22010 |  |
| Instrument | [laser](javascript:;) [scanning](javascript:;) [confocal](javascript:;)  [microscope](javascript:;) | leica | Leica TCS SP8 |  |
| Instrument | flowcytometry | Beckman | Cytoflex |  |
| Instrument | [fluorogenic](javascript:;) [quantitative](javascript:;) [PCR](javascript:;) | ABI | Quant Studio 6 |  |
| Instrument | Super sensitive automatic imaging analysis system | Protein simple | FluorChem HD2 |  |
| Instrument | spectrophotometer  microscope | Implen GmbH | NanoPhotometer 80Touch |  |
| Instrument | IVIS | PerkinElmer | IVIS^®^ Lumina III |  |
| Instrument | [Freezing](javascript:;) [microtome](javascript:;) | Leica | DM2500 |  |
| Instrument | [stereomicroscope](javascript:;) | Nikon | SMZ745T |  |
| Instrument | Cell imaging microporous plate detection system | BioTek | Cytation5 |  |
|  |  |  |  |  |
