# Supplementary material for: Adipose-derived mesenchymal stromal cells promote corneal wound healing by accelerating the clearance of neutrophils in cornea
Source: Cell Death Dis. 2020 Aug 26;11(8):707. doi: 10.1038/s41419-020-02914-y (PMC7450061; doi:10.1038/s41419-020-02914-y)
Supplement: Supplementary file 8 — Supplement Table 2 [file 41419_2020_2914_MOESM8_ESM.docx]

**Table.2. primer list**

| Primer name | Sequence (5’-3’) |
| --- | --- |
| Mouse col1a1 | TAAGGGTCCCCAATGGTGAGA  GGGTCCCTCGACTCCTACAT |
| Mouse col3a1 | ACGTAGATGAATTGGGATGCAG  GGGTTGGGGCAGTCTAGTG |
| Mouse fibronectin1 | GCTCAGCAAATCGTGCAGC CTAGGTAGGTCCGTTCCCACT |
| Mouse tnc | GAGCCCCTTTGCCTCAACAA CTTCGCCCGTGAAACCTTCTT |
| Mouse α­-sma | GTCCCAGACATCAGGGAGTAA  TCGGATACTTCAGCGTCAGGA |
| Mouse vegfa | GAGGAGCAGTTACGGTCTGTG  TCCTTTCCTTAGCTGACACTTGT |
| Mouse Mmp9 | CCACATCGAACTTCGACACTGA  TGATCTAAGCCCAGTGCATGG |
| Mouse fgf2 | TGGTGACCACAAGCTGAATG  TCCCTTGATAGACACAACTCCTC |
| Mouse ccl2 | TCTCTCTTCCTCCACCACCATG  GCGTTAACTGCATCTGGCTGA |
| Mouse cxcl2 | CCAACCACCAGGCTACAGG  GCGTCACACTCAAGCTCTG |
| Mouse cxcl5 | GTTCCATCTCGCCATTCATGC  GCGGCTATGACTGAGGAAGG |
| Mouse cxcl3 | CTGCACCCAGACAGAAGTCAT  CCGTTGGGATGGATCGCTTT |
| Mouse cxcl7 | CTCAGACCTACATCGTCCTGC  AGCGCAACAAGGATCAGGC |
| Mouse β-actin | GTGACGTTGACATCCGTAAAGA  GCCGGACTCATCGTACTCC |
| Human CD99 | AACCCACCCAAACCGATGC  TGAAAAGCTACCGGAGGAACTA |
| Human cldn5 | CTCTGCTGGTTCGCCAACAT  CAGCTCGTACTTCTGCGACA |
| Human jam-3 | TCCAGCAATCGAACCCCAG  CTTGTCTGCGAATCCGTAATGAT |
| Human vecad  Human β-actin | AAGCGTGAGTCGCAAGAATG  TCTCCAGGTTTTCGCCAGTG  TTGCCGACAGGATGCAGAAGGA  AGGTGGACAGCGAGGCCAGGAT |
|  |  |
